# Single‐Cell Profiling Reveals Distinct Immune Communication Networks in Centenarians and Elderly Controls

**DOI:** 10.1111/acel.70486

**Published:** 2026-04-14

**Authors:** Liwei Qiu, Chen Dong, Rui Zhao, Huiyuan Ye, Jian‐lin Gao, Yizhi Chen, Chi Sun, Zhifeng Gu

**Affiliations:** ^1^ Research Center of Clinical Medicine, Research Center of Gerontology and Longevity, Research Center of Immunology, Department of Rheumatology, Department of Geriatrics, Affiliated Hospital of Nantong University Nantong University Nantong China; ^2^ Department of Nephrology Hainan Hospital of Chinese PLA General Hospital, Hainan Province Chen Xiangmei Academician Team Innovation Center for Kidney Diseases Research Sanya China; ^3^ The Second School of Clinical Medicine Southern Medical University Guangzhou China

**Keywords:** centenarian, immune communication networks, immune remodeling, single‐cell transcriptome, successful aging

## Abstract

Aberrant cell–cell communication (CCC) is a recognized hallmark of aging, yet comprehensive analyses of immune CCC—particularly in the context of healthy aging—remain limited. Using single‐cell transcriptomics of PBMCs from centenarians (CEN), their offspring (CO), and elderly controls, we found that immune CCC in controls was characterized by myeloid‐derived, self‐amplifying SASP signals associated with immunosenescence and effector immune cell (EIC) exhaustion. In contrast, healthy‐aging individuals (CEN/CO) exhibited reinforced positive regulatory factor (PRF)‐mediated ligand–receptor (LR) interactions, such as IL‐15 and IL‐18, consistent with enhanced EIC cytotoxicity and immune surveillance. These findings were further supported in an independent longevity cohort. Our results suggest distinct immune CCC reprogramming with age and identify a characteristic immune remodeling pattern in centenarians, providing a framework for understanding healthy aging and exceptional longevity.

## Graphical abstract

Single‐cell profiling of PBMCs from centenarians, their offspring, and elderly controls revealed distinct myeloid‐to‐effector immune cell communication programs. General aging was characterized by SASP‐enriched signaling associated with immunosenescence, whereas healthy aging showed positive immune regulatory signaling associated with enhanced cytotoxicity and immune remodeling.

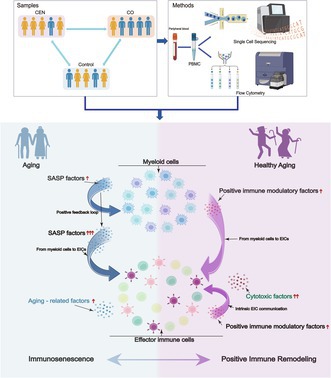

## 1 Introduction

Population aging is a global challenge, driving increased incidence of age‐related diseases and escalating healthcare costs, making the promotion of healthy aging a public health priority (Bloom et al. 2015). Aging is a principal driver of age‐related pathologies, with immunosenescence and disrupted intercellular communication recognized as central hallmarks. These processes may form a self‐perpetuating cycle: immunosenescence contributes to the accumulation of senescent cells, thereby promoting “inflammaging,” a chronic inflammatory state associated with functional decline and disease progression (Li et al. 2023; Liu et al. 2023). A key manifestation is the senescence‐associated secretory phenotype (SASP), whereby senescent cells secrete pro‐inflammatory mediators such as IL‐6, IL‐1β, and TNF‐α, which may sustain low‐grade inflammation and contribute to impaired EIC function, at least partly through disruption of CCC networks (López‐Otín et al. 2023; Franceschi et al. 2018).

Intercellular communication occurs both indirectly (e.g., via cytokines and extracellular vesicles) and directly through ligand–receptor engagement, orchestrating immune cell functions including antigen presentation, differentiation, activation, and chemotaxis, and sustaining multi‐organ immune coordination (Iba et al. 2022). Dysregulation of LR pairs—critical “molecular switches”—has been implicated in chronic inflammation, metabolic imbalance, and impaired tissue repair in aging. Key pathways involve dynamic shifts in IL‐6/IL‐6R, TNF‐α/TNFR, GPCR, Notch, and TGF‐β receptor families, which influence microenvironmental homeostasis, inflammatory responses, and regenerative capacity (Li et al. 2025).

Centenarians display unique biological features, including attenuated SASP, enhanced immune remodeling, and reduced susceptibility to age‐related disease (Santoro et al. 2021). Our previous work demonstrated that CEN and CO peripheral T cells exhibit superior cytotoxicity and immune surveillance, although the underlying CCC mechanisms remain unclear (Dong et al. 2022). Here, we applied single‐cell transcriptomics to map immune LR interaction networks in CEN, CO, and controls, with the aim of better characterizing immune communication features associated with healthy aging and exceptional longevity.

## 2 Results

### 2.1 Global Immune CCC Landscape From Single‐Cell RNA Sequencing

**FIGURE 1:**
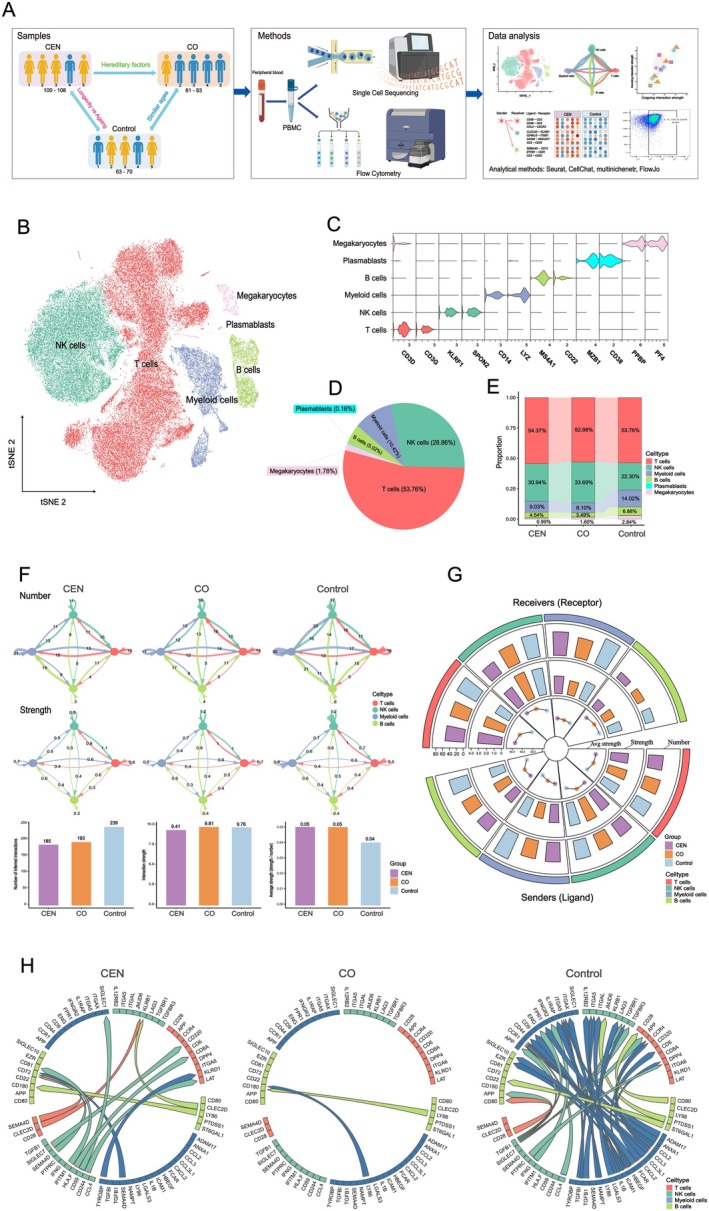
Overview of immune CCC revealed by single‐cell sequencing. (A) Schematic workflow of the study. (B) t‐SNE plot of PBMC clusters from all participants. (C) Expression profiles of canonical marker genes in each identified cluster. (D) Relative abundance of major immune cell types in the combined dataset. (E) Stacked bar plots showing cell type composition across the three groups. (F) Summary of overall CCC among four immune cell types. Top: Total CCC events per group; Middle: Total CCC strength per group; Bottom: Total CCC count, total strength, and average strength per group. (G) CCC count, total strength, and mean strength for each immune cell type acting as sender or receiver in each group. (H) Distribution of the top 50 ligand–receptor (LR) pairs ranked by CCC strength among the three groups, based on abundance‐matched repeated analyses.

We profiled PBMCs from Rugao, a well‐recognized longevity hotspot in China (centenarian prevalence ~43 per 100,000), comprising five centenarians (CEN, 100–108 years, 102.6 ± 3.4), five centenarian offspring (CO, 61–83 years, 70.8 ± 10.8), and five elderly controls living in close proximity to the centenarians and relatively age‐matched to CO (63–70 years, 67.2 ± 3.0). The mean ages of the CO and control groups were comparable, with no significant difference between them. After quality control, 87,014 cells were retained from 97,238 sequenced PBMCs (average 5801 per individual). Flow cytometry validated key CCC targets (Figure 1A).

Cells clustered into six canonical subsets: T cells, NK cells, myeloid cells, B cells, plasmablasts, and megakaryocytes (Figure 1B,C; Figure S1A) (Lyons et al. 2017). T cells predominated, followed by NK cells; plasmablasts and megakaryocytes were rare (Figure 1D; Figure S1B). Controls tended toward more myeloid cells and fewer NK cells, though without statistical significance (Figure 1E; Figure S1C).

Focusing on T, NK, myeloid, and B cells, we reconstructed CCC networks. Controls exhibited the highest number of total CCC interactions but the lowest mean communication strength (Figure 1F), a pattern consistent across both ligand‐sending and receptor‐receiving analyses. For example, in controls, NK and B cells (senders) and T and NK cells (receivers) had high interaction counts but weak signal intensity (Figure 1G). To further evaluate whether these differences were affected by unequal cell composition, we balanced the four major immune cell populations across groups and repeated the analysis 10 times (Figure S1D). Similar patterns of interaction number and interaction strength were observed after abundance matching (Figure S1E,F).

To further characterize the dominant communication signals, we analyzed the top 50 strongest LR pairs after abundance matching of the four major immune cell populations across groups and repeated analyses. Among these, 36 (72.0%) were enriched in controls, with 23 (63.9%) involving myeloid cells. Thirteen reflected intra‐myeloid SASP signaling (e.g., ICAM1, TGFB1, CCL2, CCL3, CCL3L1, CXCL2, IL1B). In contrast, CEN/CO‐enriched LR pairs predominantly linked T and NK cell CCC (Figure 1H).

### 2.2 Group‐Specific Immune CCC Patterns in the Rugao Cohort

**FIGURE 2:**
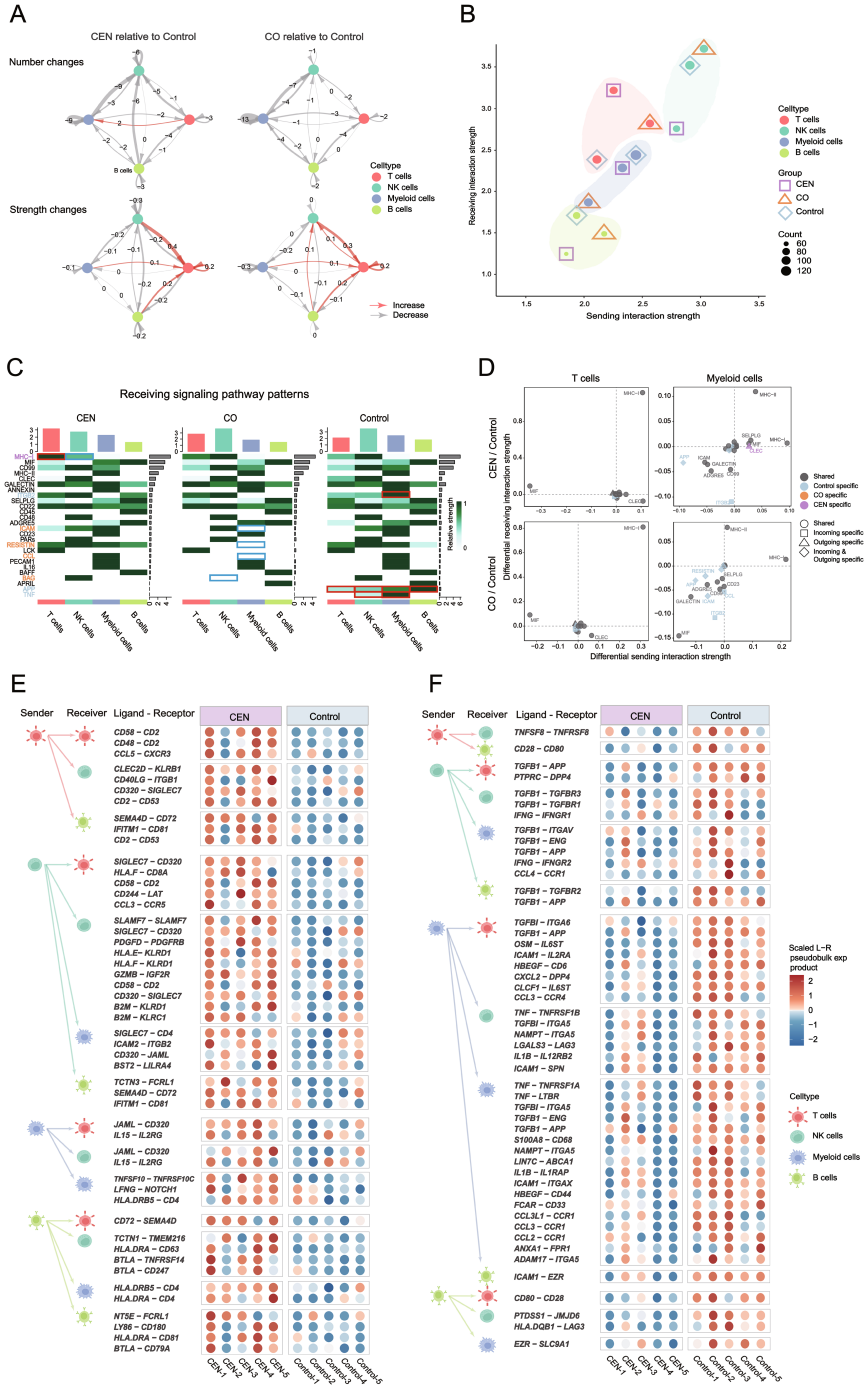
Group‐specific differences in immune CCC in the Rugao dataset. (A) Differences in CCC count and strength for the four immune cell types in CEN and CO compared with Control. (B) Two‐dimensional quadrant plot of CCC strength for each immune cell type as sender or receiver across the three groups. (C) Heatmap of pathway‐level CCC signals received by the four immune cell types. Pathway name font colors denote the group where the signal was detected. Tile borders: Blue indicates reduced or absent signals; red indicates increased or group‐specific signals. (D) Quadrant plots showing pathway‐level CCC changes in T cells and myeloid cells. Top: CEN versus Control; Bottom: CO versus Control. (E, F) Top 50 LR pairs with the largest increase in CCC strength in CEN (E) and CO (F) relative to Control. All LR pairs met *p* < 0.05.

Group comparisons revealed distinct differences in CCC event counts, signal strengths, and LR‐pair distributions. We examined: (i) intra‐subset CCC, (ii) sending versus receiving patterns, and (iii) pathway‐level variation. CEN and CO generally exhibited fewer CCC events than controls, yet their T‐cell CCC intensity—driven mainly by incoming signals—was markedly higher, explaining the lower mean CCC strength in controls (Figure 2A). A two‐dimensional strength plot identified four major trends (Figure 2B):

1. NK cells consistently had the highest communication strength; B cells the lowest.
2. CEN uniquely displayed stronger T‐cell than NK‐cell signal reception.
3. CO myeloid cells had markedly reduced CCC strength.
4. Controls showed the weakest T‐cell and strongest myeloid‐related CCC.

Pathway‐level analysis provided mechanistic context. In CEN, T cells received the strongest MHC‐I signals, whereas NK cells received the weakest (Figure 2C), reflecting differential receptor usage—T cells expressing activating CD8A/CD8B, NK cells expressing inhibitory KIRs (Figure S2A,B) (Koh et al. 2023; Lanier 2024). In CO, myeloid cells lacked ICAM and RESISTIN signaling entirely and lost BAG signaling at the ligand level (Figure 2C; Figure S2C). Controls instead showed strong ICAM and ITGB2 signaling, with unique activation of APP and TNF pathways (Figure 2C; Figure S2D), both linked to neurodegeneration and SASP activity, respectively (Vrancx and Annaert 2025; Homann et al. 2022).

Quadrant analyses comparing CEN and CO to controls revealed that CEN increases in T and NK cell reception were dominated by MHC‐I signaling, while controls exhibited heightened myeloid signaling via APP and ICAM (Figure 2D; Figure S2E). Pseudobulk LR‐pair ranking showed that CEN‐enriched pairs such as CD58–CD2, CD48–CD244, MHC‐I–CD8A, and IL‐15–IL2RG were associated with immune activation, enhancing EIC cytotoxicity and surveillance (Figure 2E). (Ho et al. 2023; Pahima et al. 2019; Aryee et al. 2022) Conversely, 64% of control‐enriched pairs were SASP‐related (e.g., TGFB1, IL1B, TNF, ICAM1), largely myeloid‐derived (59.4%) and frequently myeloid‐received (53.1%) (Figure 2F). CO versus control comparisons revealed similar patterns (Figure S2F,G).

Collectively, these results indicate that healthy‐aging individuals receive immune‐stimulatory CCC signals to sustain EIC function, whereas general aging is characterized by SASP‐driven, predominantly autocrine CCC within myeloid populations.

### 2.3 SASP‐Driven Intrinsic CCC Within Myeloid Cell Subsets in General Aging

**FIGURE 3:**
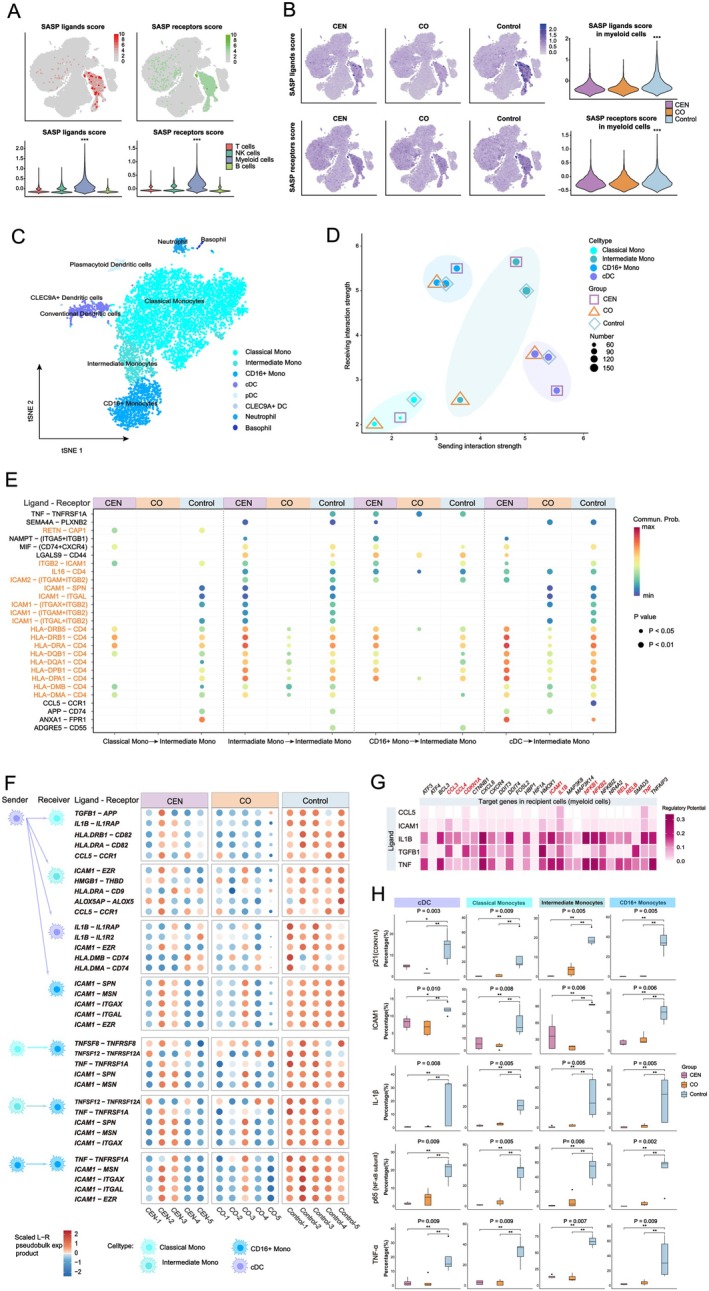
SASP‐driven intrinsic CCC among myeloid subsets in aging populations. (A) Distribution and expression levels of SASP‐related ligands and receptors across immune cell subpopulations. ***: *p* < 0.001 versus all other subpopulations. (B) Distribution and expression of SASP‐related ligands and receptors across the three groups. ***: *p* < 0.001 versus other groups. (C) t‐SNE visualization of myeloid cell subset clustering from all samples. (D) Two‐dimensional quadrant plot showing CCC strength of myeloid subsets as senders or receivers across groups. (E) Dot plot of LR pairs underlying reduced or absent incoming signals in intermediate monocytes. (F) Top five upregulated LR pairs from cDCs to the four myeloid subsets, and those received by CD16^+^ monocytes, identified in the Control group. All pairwise comparisons with CEN or CO yielded *p* < 0.05. (G) Heatmap of the top five regulatory ligands and corresponding target genes in Control‐group myeloid cells. Red‐labeled genes indicate established aging markers or SASP components. (H) Box plots showing flow cytometry–derived expression levels of key target genes in peripheral myeloid subsets across groups. *p*‐values denote overall group differences; *: *p* < 0.05, **: *p* < 0.01 for pairwise comparisons.

The preceding analyses suggested that intra‐subset, SASP‐related CCC is a hallmark of myeloid cells in the general elderly population (Control group). To validate this, we performed gene set scoring, which revealed that SASP‐associated ligands and receptors were predominantly expressed in myeloid cells, with significantly higher scores in Control compared with CEN and CO (Figure 3A,B). To further evaluate whether these differences were affected by unequal myeloid cell abundance, we performed group‐level abundance‐matched downsampling followed by repeated analyses 100 times (Figure S3A). Similar SASP ligand and receptor score patterns were observed after abundance matching, supporting the robustness of the group differences (Figure S3B–D).

Myeloid cells were further classified into eight subsets based on canonical markers (Figure 3C; Figure S3E). Among these, cDCs, classical monocytes, intermediate monocytes, and CD16^+^ monocytes were the most abundant (Figure S3F) (Lyons et al. 2017). CCC networks constructed among these four subsets showed that Control myeloid cells exhibited the highest total CCC number and strength. However, the disproportionate number of events resulted in the lowest average communication strength. In contrast, CO displayed the lowest total and average CCC strength (Figure S3G).

Two‐dimensional quadrant analysis indicated that cDCs were the most active signal senders, while intermediate and CD16^+^ monocytes were the principal signal receivers. Classical monocytes, although numerically dominant, had the weakest CCC activity. All CO myeloid subsets exhibited the lowest sending strength, and CO intermediate monocytes showed markedly reduced receiving strength compared with both CEN and Control (Figure 3D).

To investigate the cause, we analyzed LR pairs targeting intermediate monocytes. Reduced expression of integrin receptors (ITGAL, ITGAM, ITGAX) and ITGB2 in CO impaired integrin–ITGB2 heterodimer formation, critical for ICAM signaling (Bergqvist et al. 2025). Furthermore, decreased CD4 expression diminished reception of MHC‐II and IL16 signals, which are involved in antiviral responses (Figure 3E) (Forsyth and Eisenlohr 2016; Baier et al. 1995). Dot plot analysis confirmed that ITGAX, ITGAL, ITGB2, and CD4 expression was lowest in CO intermediate monocytes, while Control had the highest expression (except ITGAM, which was highest in CEN) (Figure S3H).

Given the functional significance of cDCs and CD16^+^ monocytes in CCC, we examined their LR interactions. In Control, the most highly expressed pairs were dominated by SASP ligands, including ICAM1, TGFB1, IL1B, and TNF (Figure 3F). In contrast, CEN and CO were enriched for both anti‐inflammatory pairs (e.g., HMGB1–CD163, ANXA1–FPR1, HLA‐F–LILRB1) and pro‐inflammatory ones (e.g., VCAN–TLR2, VCAN–ITGA4, ZG16B–TLR2) (Figure S4A,B) (Yang et al. 2017; Yu et al. 2025; Dulberger et al. 2017; Hope et al. 2016; Yang et al. 2025; Park et al. 2011). These findings highlight divergent intrinsic CCC programs in myeloid subsets between general aging and healthy aging.

We next examined the downstream impact of SASP‐related CCC in Control myeloid subsets. Ligands such as TNF, TGFB1, IL1B, and ICAM1 not only induced senescence markers (CDKN1A) but also amplified their own and other SASP genes, forming a self‐reinforcing autocrine loop (Figure 3G). Flow cytometry confirmed upregulation of key SASP targets—including p21, TNF‐α, IL‐1β, TGF‐β1, and NF‐κB p65—in Control myeloid cells, consistent with the single‐cell results (Figure 3H; Figure S4C), strongly supporting this model.

### 2.4 Myeloid‐Derived SASP Signals Are Associated With EIC Senescence and Exhaustion

To assess whether SASP amplification in Control myeloid subsets is associated with EIC dysfunction, we first defined EIC subsets. T cells were divided into CD8^+^ T, CD4^+^ T, Treg, NKT, MAIT T, and MALAT1^+^ T cells; NK cells into CD56^bright^, active, and adaptive NK cells, based on canonical markers (Figure S5A–D) (Lyons et al. 2017). CCC networks were then constructed between myeloid subsets and selected EIC subsets (excluding MAIT and MALAT1^+^ T cells).

**FIGURE 4:**
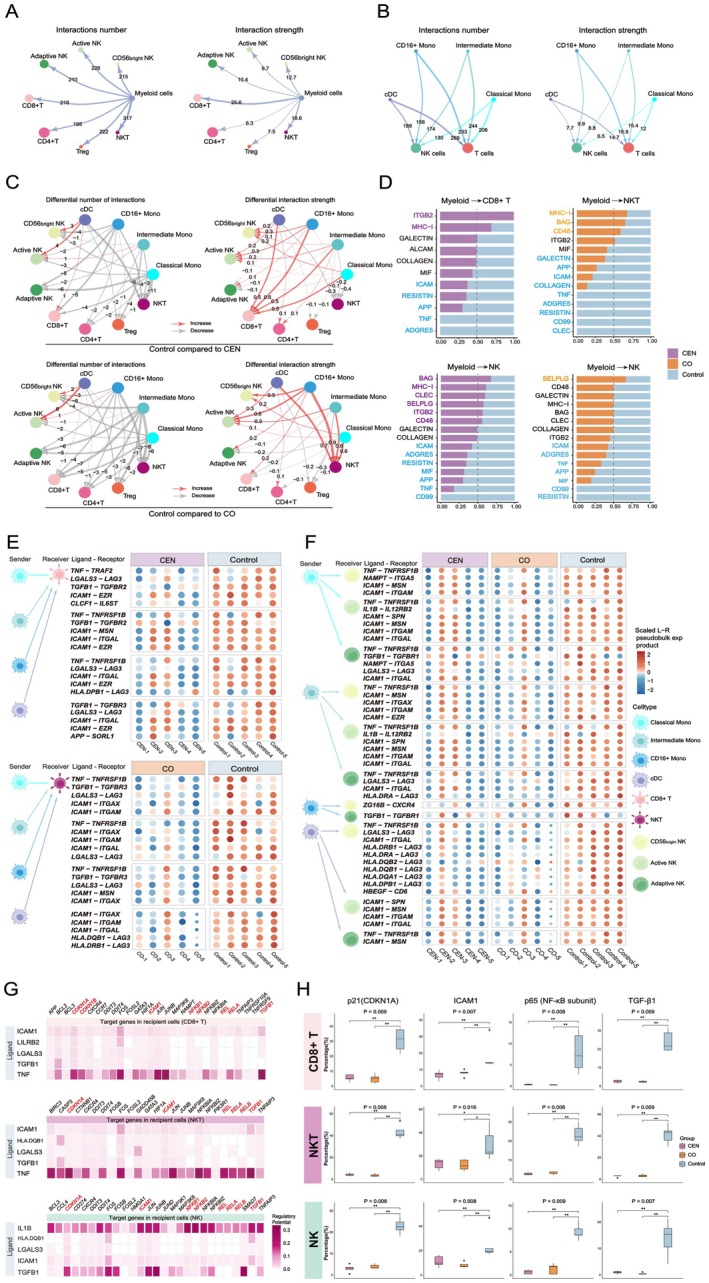
Myeloid cell–derived SASP signals promote EIC senescence and are linked to their functional exhaustion. (A) CCC counts (left) and strengths (right) from myeloid cells to EIC subsets. (B) CCC counts (left) and strengths (right) from myeloid subsets to T and NK cells. (C) Comparisons of CCC counts and strengths from myeloid subsets to EICs in Control versus CEN (top) and Control versus CO (bottom). (D) Pairwise comparisons of pathway‐level CCC strengths from myeloid cells to EICs. Pathway name font colors indicate the group in which the signal was upregulated. All inter‐group comparisons met *p* < 0.05. (E) Top: Five most upregulated LR pairs from myeloid subsets to CD8^+^ T cells in Control versus CEN. Bottom: Five most upregulated LR pairs from myeloid subsets to NKT cells in Control versus CO. All LR comparisons showed *p* < 0.05. (F) Top 50 LR pairs from myeloid subsets to NK cells upregulated in Control; all pairwise comparisons yielded *p* < 0.05. (G) In Control, the top five regulatory ligands from myeloid cells targeting CD8^+^ T cells (top), NKT cells (middle), and NK cells (bottom), with their downstream target genes. Genes in red denote established aging markers or SASP molecules. (H) Box plots showing flow cytometry–based expression of key target genes in peripheral EIC subsets from Control. *p*‐values denote overall differences; *: *p* < 0.05, **: *p* < 0.01 for pairwise comparisons.

NKT cells received the greatest number of CCC signals, whereas CD8^+^ T cells exhibited the highest signal intensity (Figure 4A). Across all groups, myeloid‐to‐T‐cell communication exceeded myeloid‐to‐NK‐cell communication. Classical monocytes, despite their abundance, contributed minimally to T‐ and NK‐cell CCC (Figure 4B).

Group‐level comparisons revealed stronger CCC from cDCs to active and CD56^bright^ NK cells in Control. Pairwise analyses showed enhanced signaling to CD8^+^ T cells in Control versus CEN, and NKT cells in Control versus CO (Figure 4C), which may reflect shifts in innate and adaptive immune balance under chronic inflammation.

Pathway analysis indicated increased ICAM, TNF, and APP signaling from myeloid cells to CD8^+^ T cells in Control versus CEN, and enrichment of GALECTIN, COLLAGEN, and other SASP‐related pathways to NKT cells in Control versus CO. For NK cells, TNF, ICAM, and APP were the predominant signaling pathways in Control versus both CEN and CO (Figure 4D).

LR‐pair analysis identified ICAM1, TGFB1, and TNF as the most prominent ligands targeting CD8^+^ T and NKT cells in Control, with TNFRSF1B (TNFR2) and TRAF2 as notable receptors. Unlike pro‐inflammatory TNFRSF1A signaling, TNF–TNFRSF1B interactions are linked to immune exhaustion (Gao et al. 2023). LAG3‐related LR pairs were significantly elevated in Control CD8^+^ T, NKT, and CD56^bright^ NK cells, and were associated with immunosuppression and chronic inflammation (Figure 4E,f; Figure S5E,F) (Graydon et al. 2020).

Analysis of exhaustion markers (PD‐1, CTLA4, TIM‐3, TIGIT) revealed markedly higher LAG3 across EIC subsets in Control, elevated TIGIT in CD8^+^ T/NKT cells, and increased HAVCR2 in NK cells. PD‐1 and CTLA4 were also elevated but remained under 10% positivity (Figure S5G). These findings suggest greater EIC exhaustion in the general aging population compared with healthy aging, although confirmation with younger controls is warranted.

Assessment of myeloid‐derived SASP ligands showed TNF as the primary regulator of CD8^+^ T and NKT cells, while IL‐1β predominantly targeted NK cells in Control. Along with ICAM1, TGFB1, and LGALS3, these ligands upregulated aging‐associated genes (CDKN1A/B, ICAM1, NFKB subunits, TGFB1) in EICs. Notably, unlike in myeloid cells, these ligands did not increase TNF expression in EICs, likely due to TNFR2‐mediated suppression, consistent with an exhaustion phenotype (Figure 4G) (Jenkins et al. 2023).

Flow cytometry confirmed elevated expression of senescence‐associated proteins (p21, TGF‐β1, p65, ICAM1) in Control EICs (Figure 4H; Figure S5H). While TGF‐β1 and NF‐κB are traditionally linked to chronic inflammation, accumulating evidence suggests they also promote EIC exhaustion under persistent inflammatory conditions (Ma et al. 2024; Fang et al. 2022).

### 2.5 EICs in Healthy Aging Populations Receive Positive CCC Signals That Enhance Cytotoxic Factor Expression

Previous studies have shown that effector immune cells (EICs) in longevity populations possess enhanced cytotoxic activity (Dong et al. 2022). However, whether this advantage is linked to distinct CCC patterns remains unclear.

**FIGURE 5:**
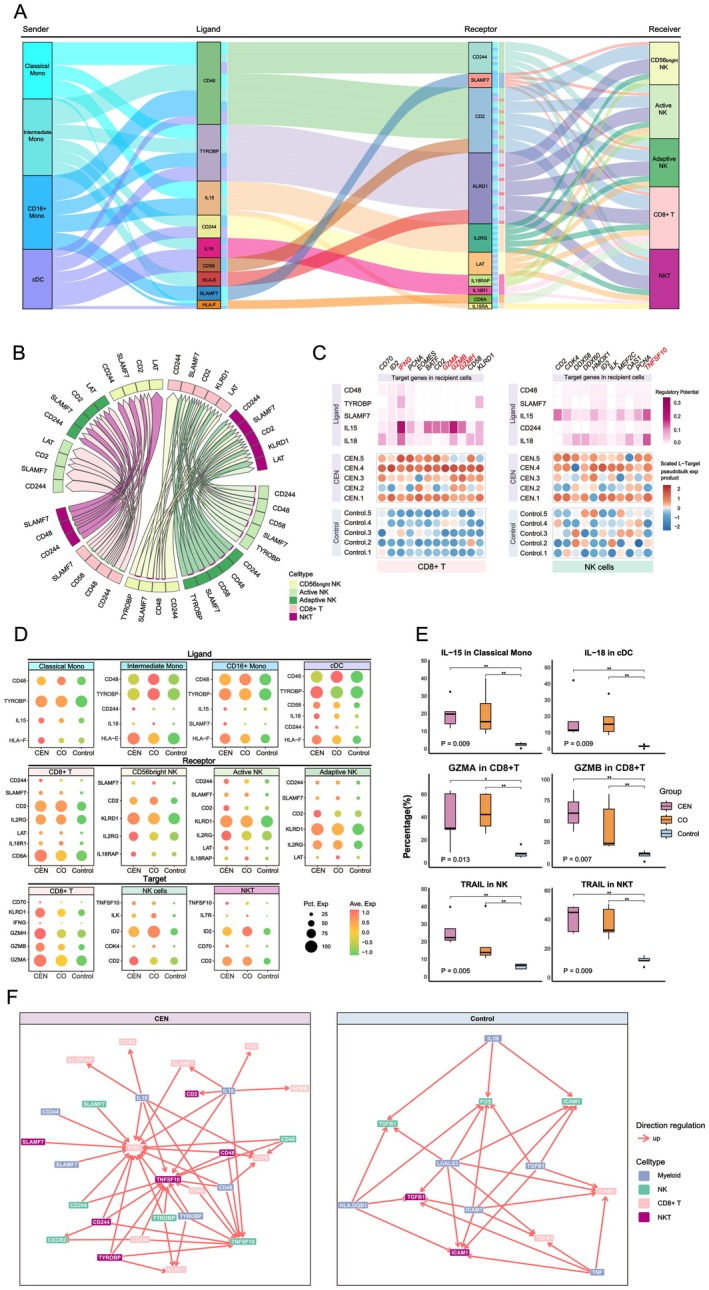
Positive CCC signals to EICs in healthy aging populations enhance cytotoxic factor expression. (A) Sankey diagram of PRF‐related LR pair networks upregulated from myeloid cells to EICs in CEN versus Control. (B) Chord diagram of upregulated intrinsic CCC‐related LR pairs among EICs in CEN versus Control. (C) Top regulatory ligands received by CD8^+^ T cells (left) and NK cells (right) in CEN, with downstream target genes. Genes in red mark well‐characterized cytotoxic effectors secreted by these cells. (D) Expression levels of selected ligands, receptors, and target genes in corresponding subsets. All genes were significantly upregulated in CEN and CO relative to Control (*p* < 0.001 for all pairwise comparisons). (E) Flow cytometry analysis of IL‐15, IL‐18, GZMA, GZMB, and TRAIL in relevant subsets. *p*‐values denote overall group differences; *: *p* < 0.05, **: *p* < 0.01 for pairwise comparisons. (F) Comparative regulatory networks of major ligands and target genes in CEN versus Control.

We first examined CCC between myeloid and EIC subsets in PBMCs. Compared with the Control group, both CEN and CO groups exhibited stronger ligand signaling from myeloid to EIC subsets, enriched in several positive regulatory factors (PRFs; e.g., IL‐15, IL‐18, CD48, HLA‐E) (Foltz et al. 2024; Park et al. 2022; Gillespie et al. 2025). Corresponding receptors for these ligands were upregulated in EIC subsets (Figure 5A). Similar CCC patterns—excluding IL‐15 and IL‐18—were also observed within EIC subsets, indicating potential internal amplification of positive signals (Figure 5B; Figure S6A,B).

To exclude the possibility that the observed group differences in PRF ligand and receptor signaling were driven by differences in cell abundance, we performed 100 rounds of random abundance‐matched downsampling for myeloid cells, CD8^+^ T cells, and NK cells across groups (Figures S3A, S6C, and S6D). We then defined the PRF ligand set in myeloid cells and the corresponding receptor sets in CD8^+^ T cells and NK cells and calculated their scores after abundance matching (Figure S6E–G). Across 100 iterations, the group differences in both PRF ligand and receptor scores remained consistent, supporting the robustness of the coordinated PRF‐related signaling patterns (Figure S6H).

Upon receiving these immunostimulatory signals, EICs markedly upregulated key cytotoxic effectors. In CD8^+^ T cells, IL‐15 and IL‐18 significantly increased expression of IFNG, GZMA, GZMB, and GZMH, with IL‐15 exerting the strongest effect. Similar trends were observed in NK and NKT cells, where the response predominantly involved TNFSF10 (TRAIL), a pro‐apoptotic mediator (Figure 5C; Figure S6I) (Smyth et al. 2003; Cardoso Alves et al. 2021). Gene expression analyses confirmed the coordinated expression of ligands, receptors, and target genes. Notably, despite significant upregulation of IFN‐γ in both CEN and CO, its proportion in CD8^+^ T cells remained low (Figure 5D; Figure S6J). Flow cytometry further validated the higher expression of PRFs (IL‐15, IL‐18) and cytotoxic effectors (GZMA, GZMB, TRAIL) in CEN and CO (Figure 5E, Figure S7D).

These ligand–receptor interactions exhibited marked selectivity and synergy. For instance, the atypical MHC‐I molecule HLA‐E, unlike classical HLA‐A, ‐B, and ‐C (aging markers), binds to KLRD1 (CD94) and forms dimers with NKG2A (KLRC1) or NKG2C (KLRC2), delivering inhibitory or stimulatory signals, respectively. The binding preference depends on TYROBP (DAP12) expression, which promotes HLA‐E–CD94/NKG2C interactions, thereby stimulating EIC proliferation, activation, and cytotoxicity when TYROBP levels are high (Braud et al. 1998; Lanier et al. 1998). Similarly, CD58 acts as a co‐stimulatory ligand via interaction with CD2, requiring the adaptor SAP (SH2D1A), whereas SHP1 and SHP2 act as negative regulators (Tangye et al. 1950; Panchal et al. 2022). Group‐wise expression analyses confirmed that these LR interactions predominantly conveyed positive immune‐regulatory CCC signals (Figure S7A–C).

Network analyses revealed clear contrasts between healthy and general aging. In centenarians, myeloid‐derived PRFs (IL‐15, IL‐18, CD48, TYROBP, SLAMF7) were associated with increased IFN‐γ, GZMB, and TRAIL expression in EICs. In contrast, general aging individuals primarily relied on SASP ligands (TGFB1, ICAM1, TNF), which reinforced senescence‐associated pathways in recipient EICs (Figure 5F; Figure S7E).

Collectively, these findings suggest a distinct immune remodeling pattern in healthy aging populations, characterized by PRF‐enriched CCC associated with EIC cytotoxicity, in contrast to the SASP‐driven CCC that predominates in general aging.

### 2.6 Validation of Immune CCC Patterns in an Independent Cohort and Integrated Summary

To further evaluate the Rugao findings, we analyzed an independent scRNA‐seq dataset from Hainan Province, China, another region with a high centenarian prevalence (13.4 per 100,000) (Yang et al. 2023). The cohort comprised three centenarians (CEN; 107–112 years, 110.3 ± 2.9) and three healthy elderly controls (Control; 73–74 years, 73.7 ± 0.6), totaling 46,958 PBMCs. Cells were categorized into major immune subsets—myeloid cells, T cells, NK cells, B cells, and megakaryocytes (Figure S8A,B)—and CCC networks were constructed among four major subsets (Figure S8C,D).

**FIGURE 6:**
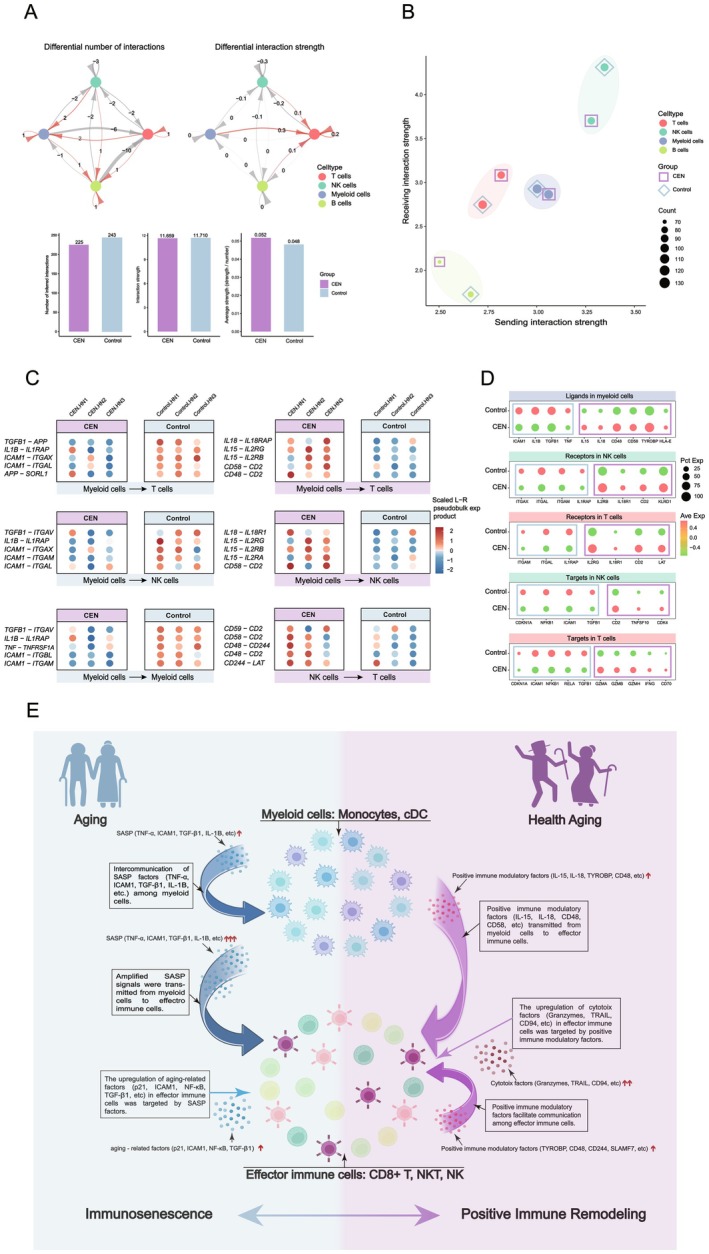
Cross‐cohort validation and study summary. (A) Top: CCC count and strength changes between groups in the HN dataset. Bottom: Combined metrics for CCC count, total strength, and mean strength. (B) Two‐dimensional quadrant plot of sending and receiving CCC strength for the four immune cell types in the HN dataset. (C) Differentially expressed LR pairs between groups. Left: SASP‐related LR pairs predominantly expressed and sent by myeloid cells in Control. Right: PRF‐related LR pairs with higher expression in CEN. All comparisons met *p* < 0.05. (D) Dot plot showing expression of key ligands, receptors, and target genes in relevant subsets. All comparisons met *p* < 0.001. (E) Schematic summarizing the major CCC features identified in general and healthy aging.

CCC trends in Hainan were broadly consistent with those observed in Rugao: the Control group exhibited more total communication events but lower mean interaction strength (Figure 6A). In CEN, T cells received more CCC signals, whereas NK cells received fewer (Figure 6B). Among the top 50 LR pairs, SASP‐associated interactions were prominent in myeloid CCC in Control, while PRF‐associated pairs (IL‐15, IL‐18, CD48, CD58, CD244) were more evident in CEN (Figure S8E).

Comparative analyses showed higher expression of SASP‐related LR pairs (TNF, TGFB1, IL1B, ICAM1) in myeloid–myeloid and myeloid–EIC interactions in Control. In contrast, CEN displayed greater expression of PRF‐related LR pairs (IL‐15, IL‐18, CD48, CD58), consistent with enhanced positive immune regulation (Figure 6C). Patterns of ligand, receptor, target gene, exhaustion marker, and selective binding protein expression were consistent with Rugao findings (Figure 6D; Figure S8F,G), suggesting broadly conserved CCC characteristics across geographically distinct aging populations.

A schematic summary (Figure 6E) summarizes the contrasting CCC features in general versus healthy aging. In general aging, SASP ligands were predominant and were associated with myeloid–myeloid autocrine loops that may amplify senescence signals and may contribute to functional decline in recipient EICs. In healthy aging, myeloid cells and other immune subsets preferentially produce PRFs (IL‐15, IL‐18, CD48, CD58, TYROBP, SLAMF7), which are associated with increased cytotoxic factor expression and EIC functional capacity. This PRF‐enriched CCC network may represent an important feature of beneficial immune remodeling in longevity populations.

## 3 Discussion

Inflammaging and immunosenescence create a self‐perpetuating cycle that accelerates biological aging and predisposes to age‐related diseases (Li et al. 2023). Although SASP components are established regulators of CCC and hallmarks of aging, their precise role in human immunosenescence, particularly in centenarians, remains poorly defined due to limited sample availability (Rodrigues et al. 2021). In this study, we systematically compared immune CCC patterns among centenarians (CEN), their offspring (CO), and general elderly populations using curated LR databases. In contrast to the CEN group, typical aging was characterized by a higher number of CCC events but lower average intensity, with prominent SASP‐driven myeloid interactions (e.g., TNF‐α, ICAM1, TGF‐β1) linked to senescence and chronic inflammation. Notably, centenarian offspring showed markedly reduced myeloid SASP signaling despite being of comparable age to the general elderly, suggesting a potential heritable or acquired advantage in immune remodeling consistent with our previous observations.

We further observed that myeloid‐derived SASP ligands were associated with EIC senescence and functional suppression via inhibitory signaling, with TNF‐α acting through distinct receptor pathways—TNFRSF1A (TNFR1) in myeloid cells and TNFRSF1B (TNFR2) in EICs (McCulloch et al. 2024; Shi and Hu 2023). TNFRSF1B‐mediated TNF‐α signaling was further linked to EIC exhaustion, as indicated by elevated LAG3, TIGIT, and HAVCR2 expression (Gao et al. 2023; Liao et al. 2023). In sharp contrast, centenarians exhibited a CCC profile favoring enhanced EIC cytotoxicity, which may involve at least two major features: (1) Receptor remodeling—increased stimulatory CD8A/B expression in T cells and reduced inhibitory KIRs in NK cells, enabling preferential recognition of protective non‐classical MHC‐I molecules (HLA‐E/F) and selective dimerization of CD94/NKG2C regulated by TYROBP (Coupel et al. 2007; Luo et al. 2023); and (2) Cytokine‐driven activation—elevated IL‐15 and IL‐18 expression in myeloid cells, which was associated with increased expression of cytotoxic effectors such as granzymes and TRAIL in EICs (Guo et al. 2025; Kayagaki et al. 1950). IL‐15 and IL‐18 are well‐documented for enhancing metabolic activation, memory persistence, immune repair, and anti‐exhaustion in cancer immunotherapy (Foltz et al. 2024; Tarannum and Romee 2021; Jaspers et al. 2023) and their synergy is known to augment NK cell memory, cytotoxicity, and lifespan while downregulating KIR expression, consistent with our findings (Sivori et al. 2020). Together, these features may help explain the preserved immune competence and positive immune remodeling observed in exceptional longevity.

Our results highlight elevated myeloid‐derived IL‐15 and IL‐18 as candidate CCC mediators associated with enhanced EIC cytotoxicity in healthy aging. Multiple PRFs likely act synergistically; for example, CD48–CD244 interactions require the adaptor SAP (SH2D1A) for activation, while SHP1/SHP2 mediate inhibitory control (Waggoner and Kumar 2012; Taylor et al. 2009). This PRF‐mediated “switch‐like” CCC architecture in centenarians may help maintain immune function, sustain cytotoxicity, limit exhaustion, and contribute to protection against malignancy and persistent infections. Importantly, abundance‐matched repeated analyses supported the robustness of the major SASP‐ and PRF‐related CCC patterns. These findings were further supported by analysis of an independent longevity cohort and flow cytometry validation.

Nonetheless, certain limitations must be acknowledged. First, the sample size was relatively small, and younger control groups were not included. Although abundance‐matched repeated analyses supported the major SASP‐ and PRF‐related CCC patterns, some comparisons may still be sensitive to cohort size and warrant validation in larger cohorts. In addition, the unequal sex distribution among groups may represent a potential source of bias. Moreover, functional validation of the inferred LR interactions remains limited. Reliance on currently available LR databases, unexpectedly low IFN‐γ levels in centenarians despite strong cytotoxic profiles, and insufficient characterization of B‐cell–mediated CCC warrant further investigation.

In summary, this study provides a network‐level framework for understanding how intercellular communication shapes immunosenescence and aging trajectories. SASP‐associated CCC was linked to immune dysfunction in typical aging, whereas centenarians exhibited a PRF‐enriched CCC landscape associated with enhanced immune resilience and positive remodeling. These findings highlight candidate CCC pathways that may be relevant to healthy aging interventions and future mechanistic studies.

## 4 Experimental Section

### 4.1 Experimental Design

This study investigated immune cell–cell communication (CCC) features associated with longevity and healthy aging. Centenarians (CEN) provide a unique model for elucidating anti‐tumor, anti‐infection, and aging‐resistance mechanisms, yet their immune CCC landscape remains largely unexplored. To account for potential genetic and environmental influences, we recruited individuals from CEN, their offspring (CO), and elderly controls living in close proximity to the centenarians (Control) from the longevity hotspot, Rugao, China. Peripheral blood mononuclear cells (PBMCs) from 15 participants (5 per group) underwent single‐cell RNA sequencing (scRNA‐seq) and integrative bioinformatics analyses to characterize CCC patterns. Key findings were further evaluated in an independent validation cohort from Sanya, Hainan.

### 4.2 Subjects and Samples

The CEN group included healthy centenarians identified through comprehensive clinical evaluation. The CO group consisted of healthy offspring living with the centenarians, and the same health criteria were applied to this group. The control group included elderly individuals living in close proximity to the centenarians and relatively age‐matched to the CO group in order to reduce potential confounding from environmental and lifestyle‐related factors. Morning venous blood was collected in EDTA or heparin tubes. A comprehensive clinical evaluation, including a complete blood count, metabolic panel, liver and kidney function tests, fasting glucose, lipid profile, and blood pressure, confirmed the absence of overt disease. Detailed blood pressure and biochemical parameters for all participants from both the discovery and validation cohorts are provided in Supporting Information S1. PBMCs were isolated within 4 h using Ficoll‐Paque PLUS density gradient centrifugation, followed by two PBS washes. Cell viability and counts were assessed before downstream applications.

### 4.3 Independent Validation Cohort

An independent validation cohort was recruited from Sanya, Hainan, China, and included three healthy centenarians and three elderly controls. The inclusion criteria were the same as those used for the Rugao cohort. Peripheral blood samples were collected and processed using the same procedures as described above. PBMCs from this cohort also underwent single‐cell RNA sequencing. Clinical and biochemical information for the validation cohort is included in Supporting Information S1.

### 4.4 Single‐Cell RNA Sequencing

Single‐cell libraries were prepared using a 10× Genomics Chromium Single Cell 3′ Reagent Kit v3. PBMCs were encapsulated with barcoded gel beads to form Gel Bead‐in‐Emulsions (GEMs). After reverse transcription and PCR amplification of full‐length cDNA, sequencing libraries were constructed for both 5′ and 3′ ends. Sequencing was performed on the Illumina HiSeq 4000 platform.

### 4.5 Flow Cytometry Analysis

PBMCs, prepared as described above, were stained with fluorochrome‐conjugated antibodies targeting key markers for myeloid, T, and NK cell subsets (Table S1; Supporting Information). Data were acquired on a BD LSRFortessa flow cytometer and analyzed using FlowJo v9.

### 4.6 Bioinformatics Analysis

Analyses were performed in R (v4.4.0). Following doublet removal with the scDblFinder package (Germain et al. 2020), data were processed in Seurat (v4.4.0) for normalization, scaling, batch correction, clustering, and differential expression analysis (Satija et al. 2015). CCC network inference, interaction strength quantification, and pathway analysis were performed using CellChat (v1.6.1) (Jin et al. 2021). Group‐specific ligand–receptor (LR) pair profiling and target prediction were conducted with multinichenetr (v1.0.3) (Browaeys et al. 2020).

### 4.7 Abundance‐Matched Downsampling Analysis

To minimize the influence of unequal cell composition, abundance‐matched downsampling was applied in selected robustness analyses. Cells from the relevant cell populations were randomly subsampled to matched numbers across groups. For each comparison, the matched cell number was set to 80% of the smallest available cell count among groups to avoid fixing the group with the lowest cell number across all iterations and to preserve stochasticity in each repetition. Each repetition consisted of a new round of random downsampling followed by the corresponding downstream analysis on the newly generated matched dataset. The iteration numbers and analysis endpoints for each setting are described in the corresponding Results sections.

### 4.8 Statistical Analysis

Gene expression differences were assessed with the Wilcoxon test (FindMarkers, Seurat). LR interaction strength was calculated as the product of pseudobulk ligand and receptor expression levels. Dot plots were generated for LR pairs meeting *p* < 0.05 and logFC > 0.50. For three‐group comparisons, Kruskal–Wallis tests were used, followed by pairwise Mann–Whitney *U* tests with Bonferroni correction when significant. A two‐tailed *p* < 0.05 was considered statistically significant.

## Author Contributions

*Conceptualization*: Liwei Qiu, Chen Dong, Chi Sun, and Zhifeng Gu. *Data curation*: Chen Dong, Chi Sun, and Yizhi Chen. *Formal analysis*: Liwei Qiu and Chen Dong. *Funding acquisition*: Liwei Qiu, Chen Dong, Jian‐lin Gao, and Zhifeng Gu. *Investigation*: Chen Dong, Rui Zhao, and Yizhi Chen. *Methodology*: Liwei Qiu, Yizhi Chen, and Zhifeng Gu. *Project administration*: Yizhi Chen, Chi Sun, and Zhifeng Gu. *Resources*: Jian‐lin Gao, Yizhi Chen, Chi Sun, and Zhifeng Gu. *Software*: Liwei Qiu and Huiyuan Ye. *Supervision*: Chen Dong and Rui Zhao. *Validation*: Chen Dong and Huiyuan Ye. *Visualization*: Liwei Qiu. *Writing – original draft*: Liwei Qiu and Chen Dong. *Writing – review and editing*: Chen Dong, Chi Sun, and Zhifeng Gu.

## Funding

This work was supported by National Natural Science Foundation of China, Grant/Award Number: 92574113, U23A20470 and 82502178; Jiangsu Provincial Preventive Medicine Research Project, Grant/Award Number: Ym2023112; Science and technology Project of Nantong City, Grant/Award Number: XNBH00031655; Jiangsu Provincial Medical Key Discipline Cultivation Unit, Grant/Award Number: JSDW202205; China Higher Education Institution Industry‐University‐Research Innovation Fund, Grant/Award Number: 2023HT041; Jiangsu Provincial Research Hospital, Grant/Award Number: YJXYY202204; Jiangsu Funding Program For Excellent Postdoctoral Talent.

## Disclosure

Figure Illustration Source: All schematic and cellular illustrations were created in BioRender. Qiu, L.(2026) http://Biorender.com/v34valv and are reproduced with permission.

## Ethics Statement

The study was approved by the Ethics Committee of Affiliated Hospital of Nantong University (approval number 2019‐K045).

## Consent

Written informed consent was obtained from all participants prior to inclusion in the study.

## Conflicts of Interest

The authors declare no conflicts of interest.

## Supporting information


**Figure S1:** Immune cell composition and abundance‐matched robustness analyses of global CCC patterns. (A) UMAP visualization of PBMC clusters from all samples. (B) Counts and relative proportions of each immune cell subset.
(C) Immune cell composition in individual samples. (D) Numbers of the four major immune cell populations (T cells, NK cells, myeloid cells, and B cells) across the three groups before and after abundance matching. (E) Total number of inferred CCC interactions across the three groups in 10 abundance‐matched repeated analyses. (F) Total strength of inferred CCC interactions across the three groups in 10 abundance‐matched repeated analyses. (G) Average interaction strength across the three groups in 10 abundance‐matched repeated analyses.
**Figure S2:** Receptor remodeling and differential CCC signaling patterns in effector immune cells across aging groups.
(A) Expression of MHC class I co‐receptors CD8A and CD8B in CD8^+^ T cells. (B) Expression of MHC class I inhibitory receptors (KIRs) in NK cells. (C) Pathway signaling strength transmitted by the four major immune cell types in each group. Pathway name font color denotes the originating group. Tile borders indicate decreased/absent (blue) or increased/group‐specific (red) signals.
(D) Aggregate signal strength of key pathways across the four immune cell types and three groups, following annotation conventions in (F). (E) Quadrant plot comparing NK cell communication strength in the CEN group versus Control.
(F, G) Top 50 ligand–receptor (LR) pairs with the largest CCC strength increase in CO versus Control (F) and Control versus CO (G), all with *p* < 0.05. Asterisks in (A and B) denote significance relative to Control: *p <* 0.05 (*), *p* < 0.01 (**), *p* < 0.001 (***).
**Figure S3:** Myeloid subset features and abundance‐matched robustness analyses of SASP‐related intrinsic CCC. (A) Numbers of myeloid cells across the three groups before and after abundance matching. (B) SASP ligand scores in myeloid cells across the three groups in 10 representative analyses randomly selected from 100 abundance‐matched repeated analyses. (C) SASP receptor scores in myeloid cells across the three groups in 10 representative analyses randomly selected from 100 abundance‐matched repeated analyses. (D) Counts of 100 abundance‐matched repeated analyses with *p* < 0.05 for CEN versus Control and CO versus Control comparisons of SASP ligand and receptor scores in myeloid cells. (E) Expression of canonical marker genes across myeloid subsets.
(F) Counts and proportions of myeloid cell subsets. (G) CCC networks among myeloid subsets: total CCC counts (top), total CCC strength (middle), and combined summary metrics (bottom). (H) Expression of receptors associated with lost or attenuated signaling in intermediate monocytes. Asterisks in (B, C, and H) denote significance relative to the other two groups: *p < 0.05, **p < 0.01, ***p < 0.001.
**Figure S4:** Divergent intrinsic CCC programs and flow cytometric validation of SASP‐related features in myeloid subsets. (A, B) Upregulated LR pairs in cDCs, intermediate monocytes, and CD16^+^ monocytes in CEN (A) and CO (B) versus Control, all with *p* < 0.05.
(C) Representative flow cytometry plots showing key target gene expression in myeloid cells from the Control group. Each panel includes: top left, gating strategy; top right, representative CEN sample; bottom left, representative CO sample; bottom right, representative Control sample.
**Figure S5:** SASP‐related CCC from myeloid cells to EICs in aging population. (A) t‐SNE plot of T cell clusters. (B) Marker gene expression in T cell subsets. (C) t‐SNE plot of NK cell clusters. (D) Marker gene expression in NK cell subsets. (E) LR pairs upregulated in CD8^+^ T cells receiving SASP‐ or LAG3‐related signals from myeloid subsets in CEN versus Control (*p* < 0.05 for all). (F) LR pairs upregulated in NKT cells receiving SASP‐ or LAG3‐related signals from myeloid subsets in CO versus Control (*p* < 0.05 for all). (G) Expression of exhaustion markers in EICs, with significance relative to Control: *p* < 0.05 (*), *p* < 0.01 (**), *p* < 0.001 (***). (H) Flow cytometry sorting plots of major upregulated target genes in EICs from the Control group, arranged as in Figure S4C.
**Figure S6:** PRF‐related ligand–receptor features and abundance‐matched robustness analyses supporting enhanced EIC signaling in healthy aging. (A, B) Upregulated LR pairs in CO versus Control: (A) from NK subsets to CD8^+^ T and NKT cells; (B) from CD8^+^ T and NKT cells to NK subsets. (C) Numbers of CD8^+^ T cells across the three groups after group‐level abundance matching.
(D) Numbers of NK cells across the three groups after group‐level abundance matching. (E) PRF ligand scores in myeloid cells across the three groups in 10 representative analyses randomly selected from 100 abundance‐matched repeated analyses. (F) PRF receptor scores in CD8^+^ T cells across the three groups in 10 representative analyses randomly selected from 100 abundance‐matched repeated analyses. (G) PRF receptor scores in NK cells across the three groups in 10 representative analyses randomly selected from 100 abundance‐matched repeated analyses. (H) Counts of 100 abundance‐matched repeated analyses with *p* < 0.05 for CEN versus Control and CO versus Control comparisons of PRF ligand scores in myeloid cells and PRF receptor scores in CD8^+^ T cells and NK cells. (I) Key regulatory ligands and downstream targets received by NKT cells in healthy aging; red‐labeled targets indicate known cytotoxic effectors. (J) Expression of key ligands, receptors, and target genes in relevant subsets. Asterisks in (E, F, G and J) denote significance relative to the Control: *p < 0.05, **p < 0.01, ***p < 0.001.
**Figure S7:** Selective PRF‐related ligand–receptor features and downstream cytotoxic validation in effector immune cells. (A) Expression of MHC‐I molecules in myeloid cells. (B, C) Expression of selected receptor genes in NK cells (B) and T cells (C). (D) Flow cytometry plots of IL‐15, IL‐18, GZMA, GZMB, and TRAIL in relevant subsets, arranged as in Figure S2G. (E) Network diagram of LR pairs upregulated in CO versus Control. Asterisks in (A–C) denote significance relative to the Control: *p < 0.05, **p < 0.01, ***p < 0.001.
**Figure S8:** Validation in the Hainan dataset. (A) t‐SNE visualization of PBMC clusters in the Hainan dataset. (B) Marker gene expression in identified subpopulations. (C, D) Total CCC counts (C) and overall CCC strength (D) in CEN versus Control (Hainan dataset). (E) Top 50 LR pairs ranked by interaction strength in the Hainan dataset. (F) Expression of MHC‐I molecules in myeloid cells (*p* < 0.001 for all group comparisons). (G) Top: exhaustion marker expression in T and NK cells. Bottom: expression of selected receptor genes in T and NK cells (*p* < 0.001 for all group comparisons).
**Table S1:** List of antibodies and fluorochromes used for flow cytometry.

## Data Availability

All data needed to evaluate the conclusions in the paper are present in the paper and/or the Supporting Information.
