# Supplementary material for: Single‐Cell Profiling Reveals Distinct Immune Communication Networks in Centenarians and Elderly Controls
Source: Aging Cell. 2026 Apr 14;25(4):e70486. doi: 10.1111/acel.70486 (PMC13078127; doi:10.1111/acel.70486)
Supplement: Supplementary file 1 — Figure S1: Immune cell composition and abundance‐matched robustness analyses of global CCC patterns. (A) UMAP visualization of PBMC clusters from all samples. (B) Counts and relative proportions of each immune cell subset. (C) Immune cell composition in individual samples. (D) Numbers of the four major immune cell populations (T cells, NK cells, myeloid cells, and B cells) across the three groups before and after abundance matching. (E) Total number of inferred CCC interactions across the three groups in 10 abundance‐matched repeated analyses. (F) Total strength of inferred CCC interactions across the three groups in 10 abundance‐matched repeated analyses. (G) Average interaction strength across the three groups in 10 abundance‐matched repeated analyses. Figure S2: Receptor remodeling and differential CCC signaling patterns in effector immune cells across aging groups. (A) Expression of MHC class I co‐receptors CD8A and CD8B in CD8+ T cells. (B) Expression of MHC class I inhibitory receptors (KIRs) in NK cells. (C) Pathway signaling strength transmitted by the four major immune cell types in each group. Pathway name font color denotes the originating group. Tile borders indicate decreased/absent (blue) or increased/group‐specific (red) signals. (D) Aggregate signal strength of key pathways across the four immune cell types and three groups, following annotation conventions in (F). (E) Quadrant plot comparing NK cell communication strength in the CEN group versus Control. (F, G) Top 50 ligand–receptor (LR) pairs with the largest CCC strength increase in CO versus Control (F) and Control versus CO (G), all with p < 0.05. Asterisks in (A and B) denote significance relative to Control: p < 0.05 (*), p < 0.01 (**), p < 0.001 (***). Figure S3: Myeloid subset features and abundance‐matched robustness analyses of SASP‐related intrinsic CCC. (A) Numbers of myeloid cells across the three groups before and after abundance matching. (B) SASP ligand scores in myeloid cells acros [file ACEL-25-e70486-s001.zip › supporting information.docx]

**Figures with Supplementary Information**

**Figure S1**

**
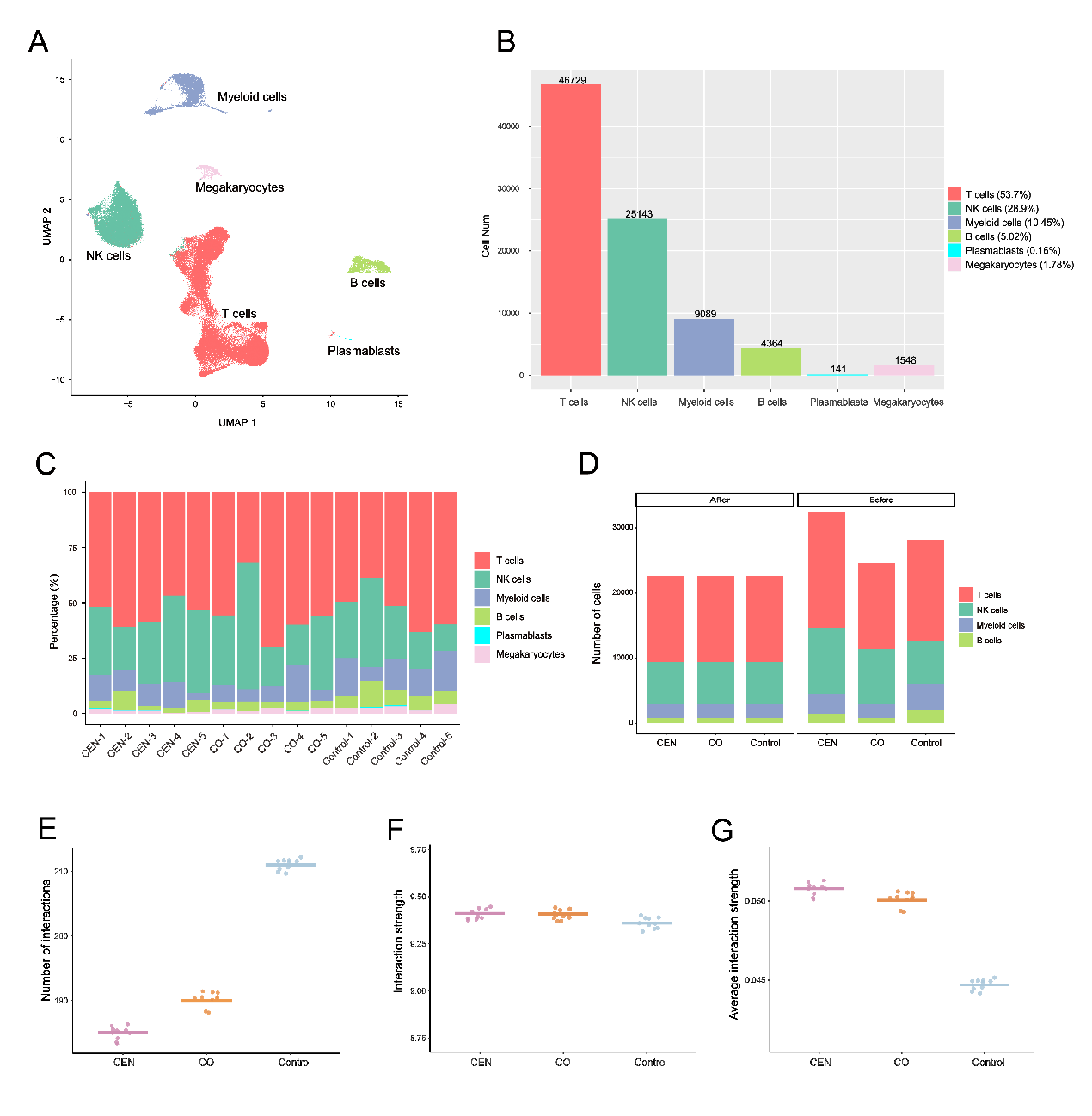
**

**Figure S2**

**
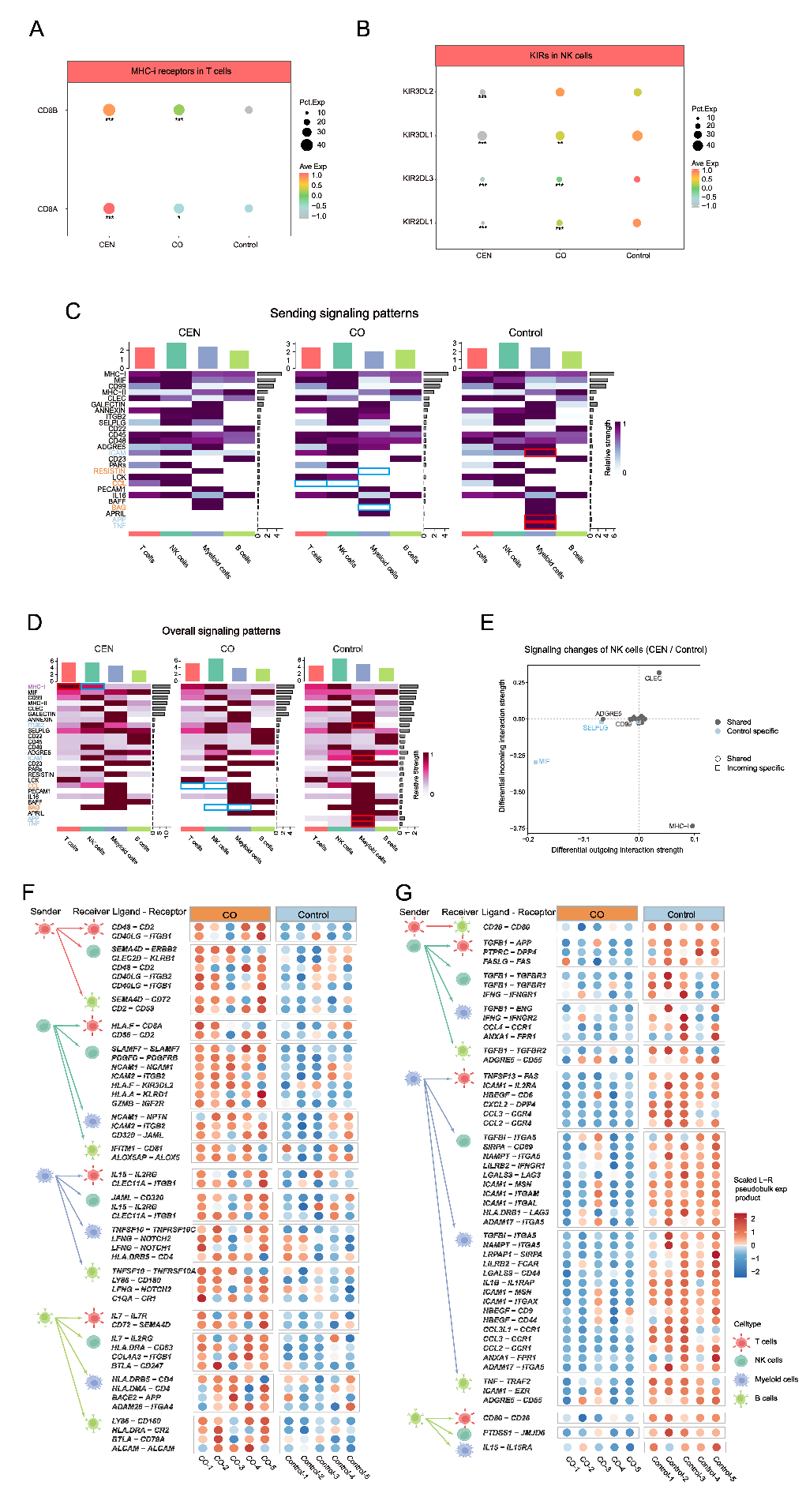
**

**Figure S3**

**
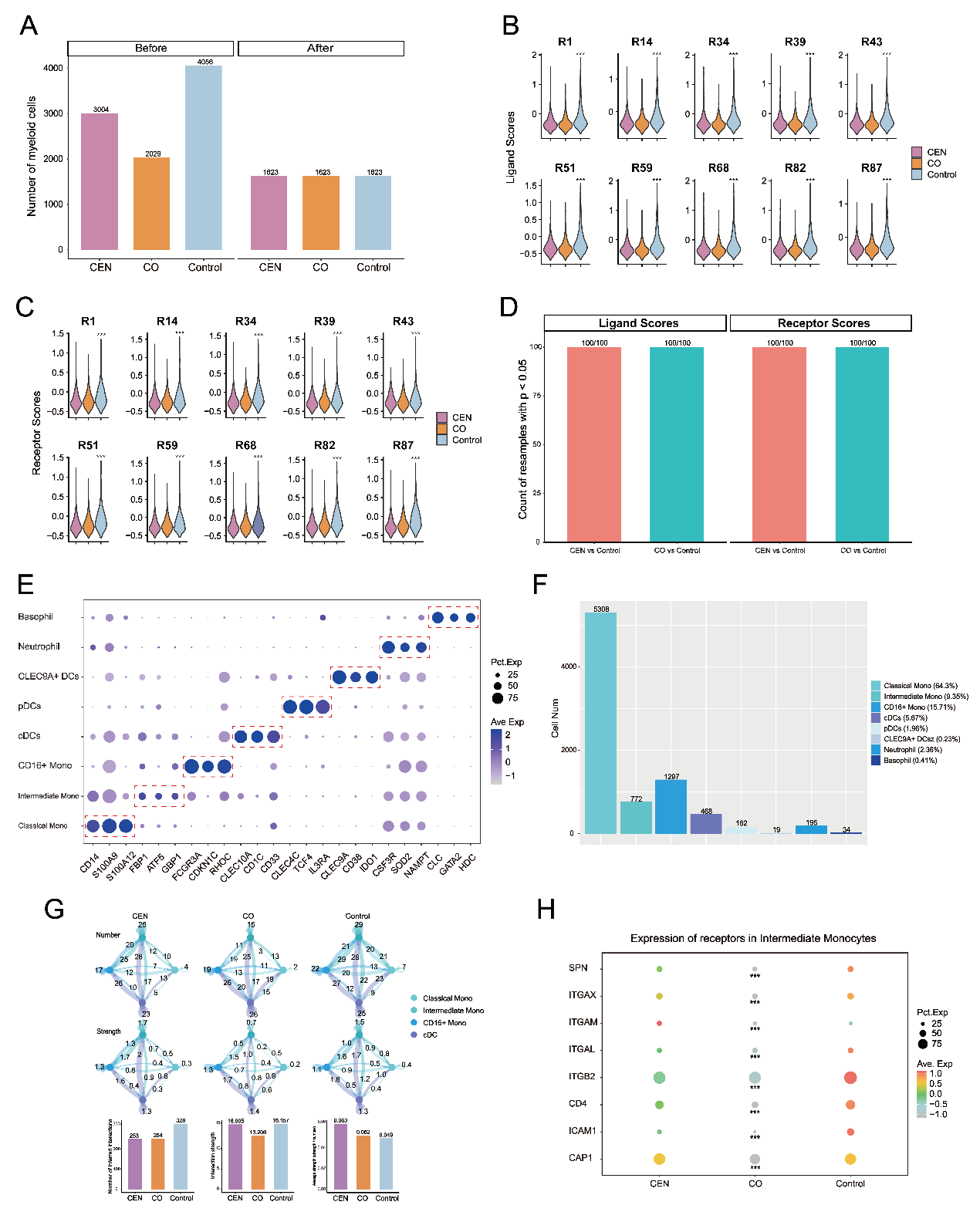
**

**Figure S4**

**
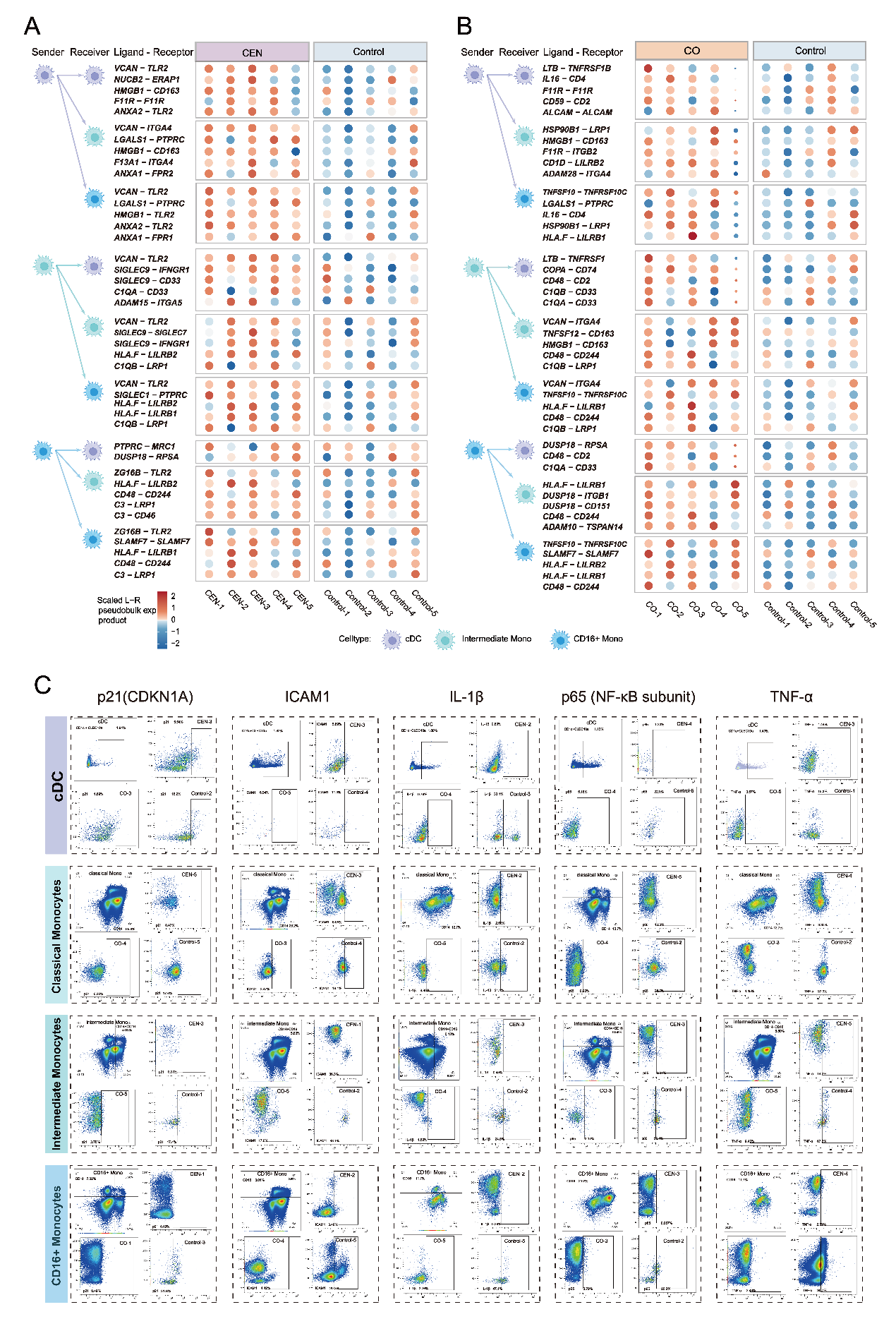
**

**Figure S5**

**
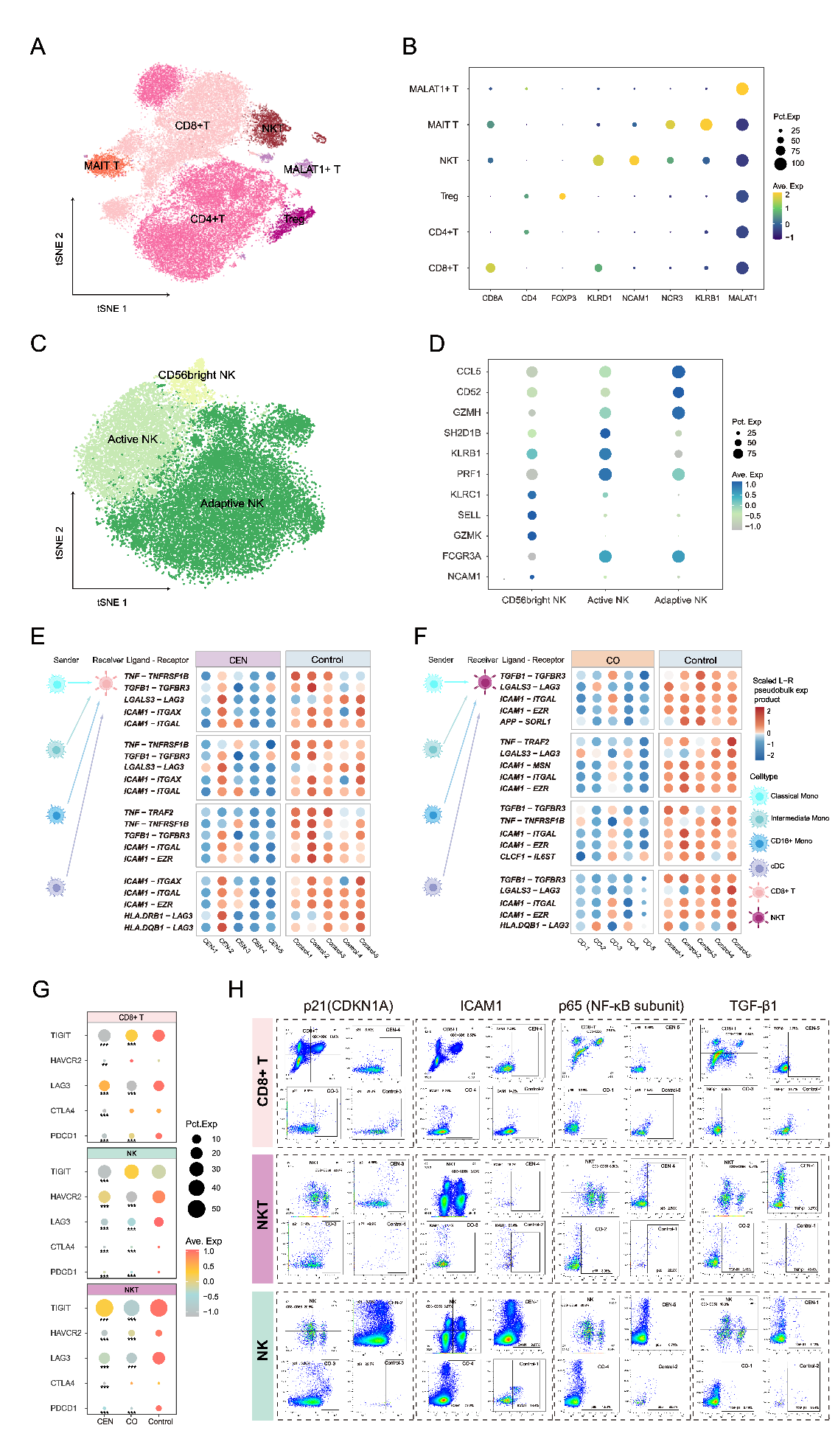
**

**Figure S6**

**
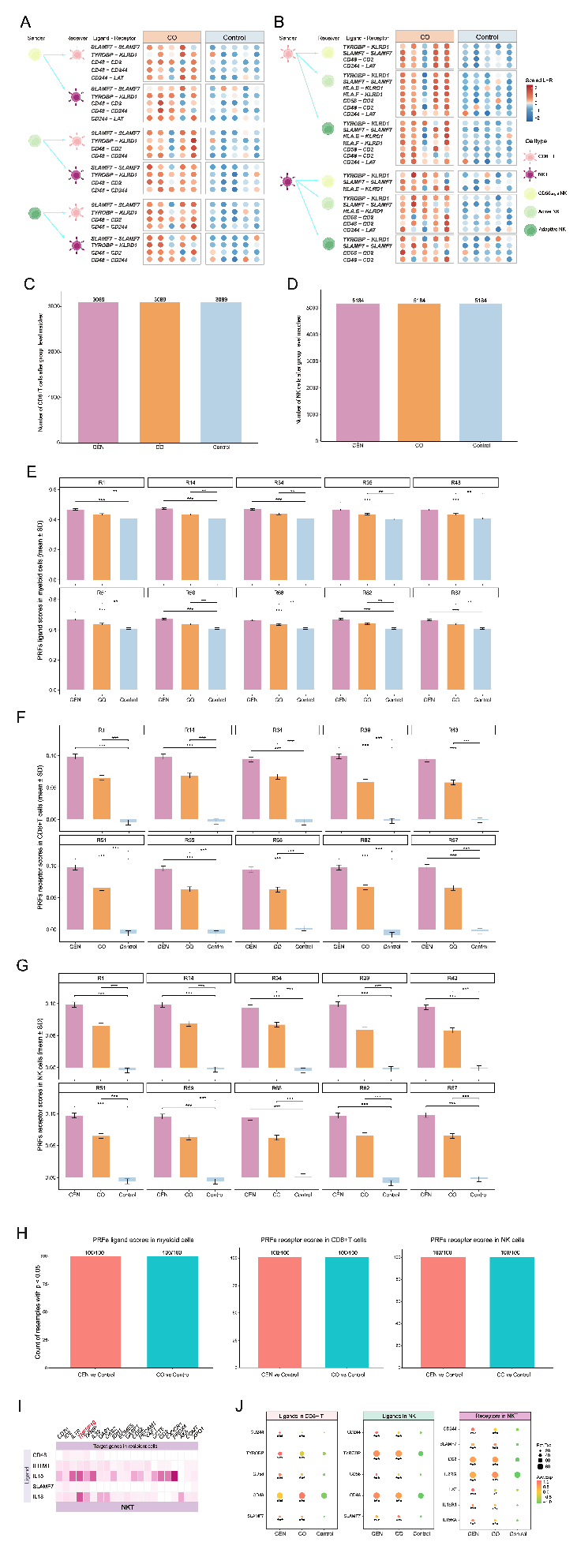
**

**Figure S7**

**
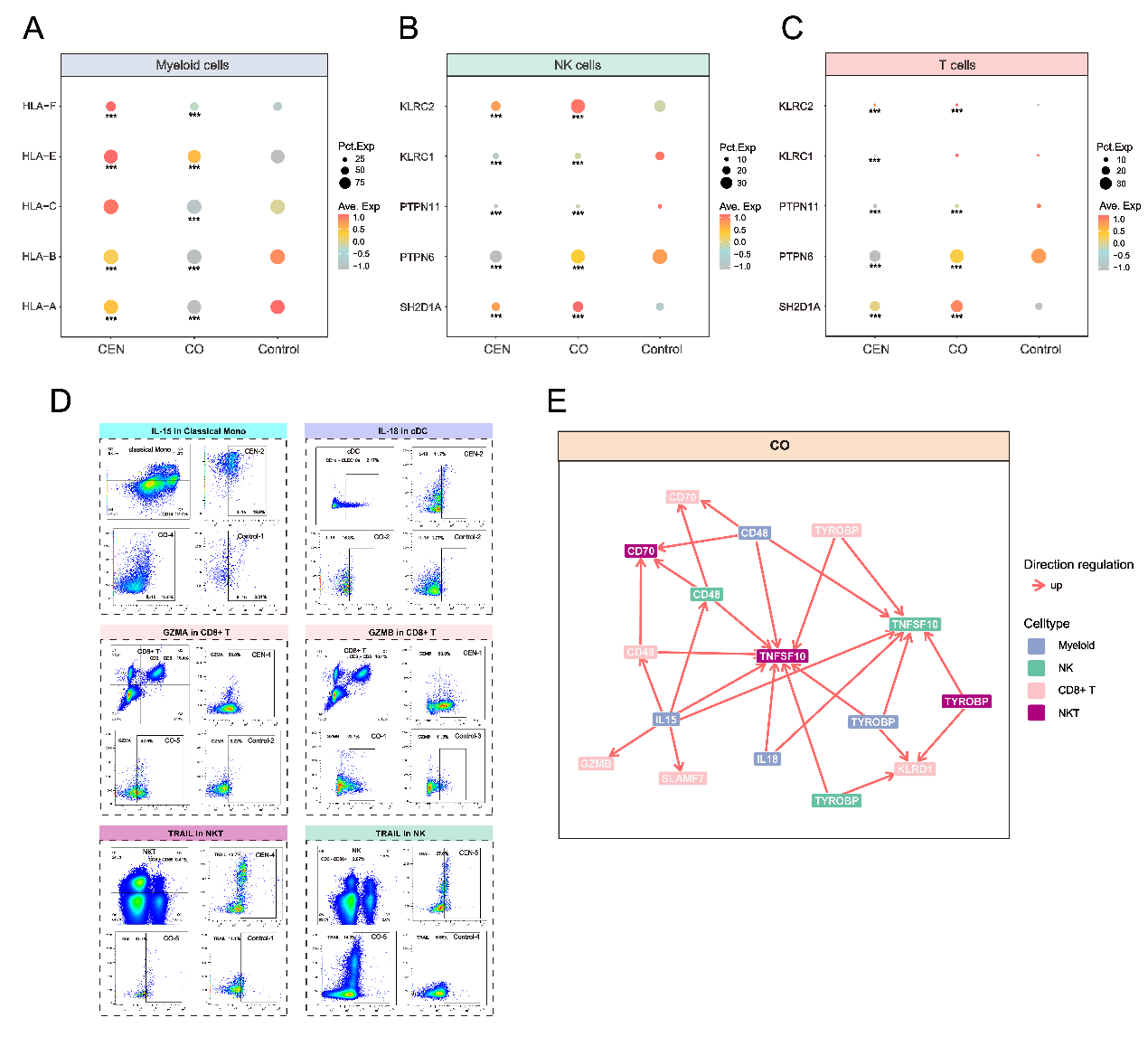
**

**Figure S8**

**
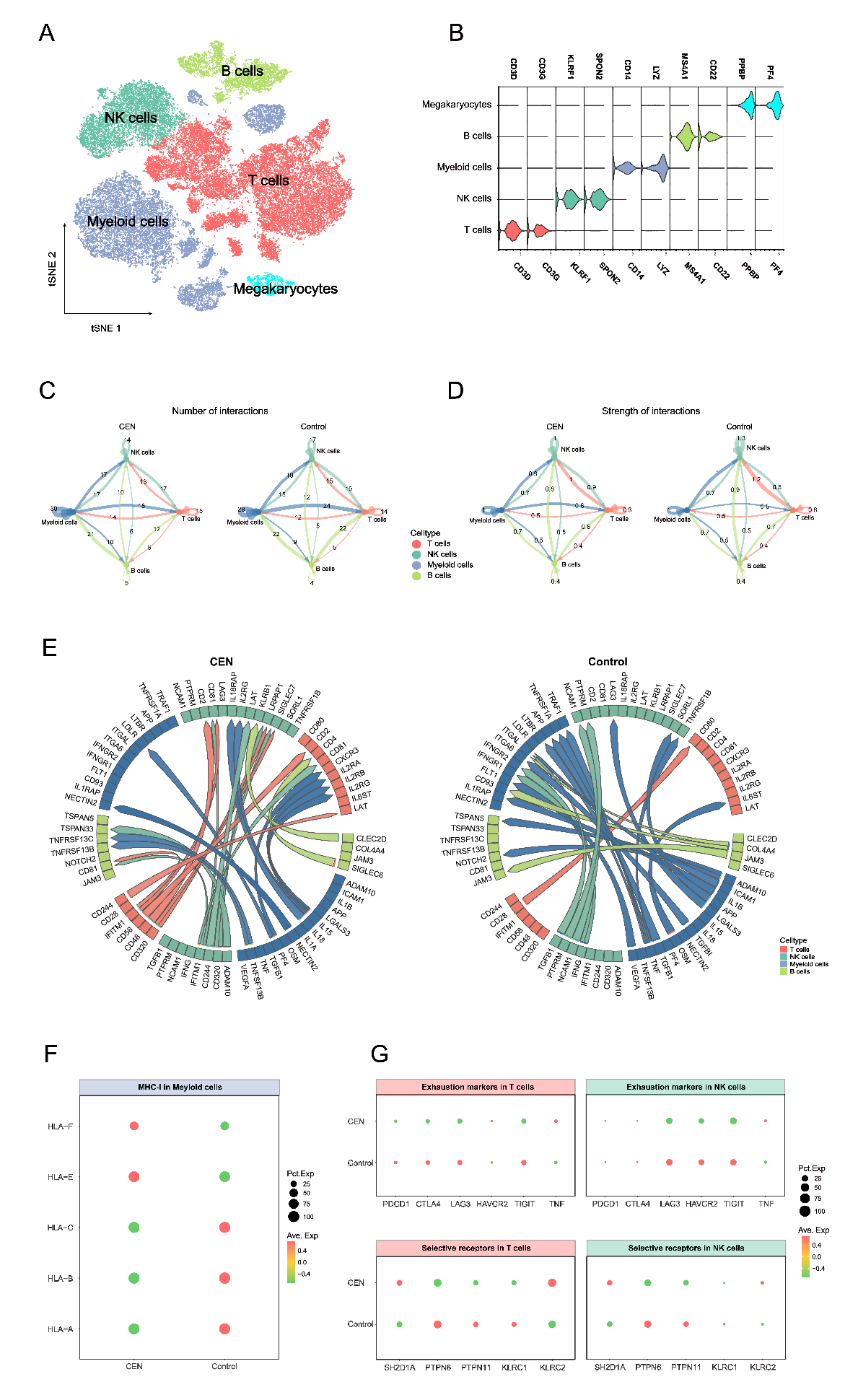
**

**Supplement Figure Legends**

**Figure S1. Immune cell composition and abundance-matched robustness analyses of global CCC patterns.**

(A) UMAP visualization of PBMC clusters from all samples.

(B) Counts and relative proportions of each immune cell subset.

(C) Immune cell composition in individual samples.

(D) Numbers of the four major immune cell populations (T cells, NK cells, myeloid cells, and B cells) across the three groups before and after abundance matching.
(E) Total number of inferred CCC interactions across the three groups in 10 abundance-matched repeated analyses.
(F) Total strength of inferred CCC interactions across the three groups in 10 abundance-matched repeated analyses.
(G) Average interaction strength across the three groups in 10 abundance-matched repeated analyses.

**Figure S2. Receptor remodeling and differential CCC signaling patterns in effector immune cells across aging groups.**

(A) Expression of MHC class I co-receptors CD8A and CD8B in CD8⁺ T cells.

(B) Expression of MHC class I inhibitory receptors (KIRs) in NK cells.

(C) Pathway signaling strength transmitted by the four major immune cell types in each group. Pathway name font color denotes the originating group. Tile borders indicate decreased/absent (blue) or increased/group-specific (red) signals.

(D) Aggregate signal strength of key pathways across the four immune cell types and three groups, following annotation conventions in (F).

(E) Quadrant plot comparing NK cell communication strength in the CEN group versus Control.

(F, G) Top 50 ligand–receptor (LR) pairs with the largest CCC strength increase in CO *vs*. Control (F) and Control *vs*. CO (G), all with *P* < 0.05.

Asterisks in (A) and (B) denote significance relative to Control: *P <* 0.05 (*), *P* < 0.01 (**), *P* < 0.001 (***)*.*

**Figure S3. Myeloid subset features and abundance-matched robustness analyses of SASP-related intrinsic CCC.**

(A) Numbers of myeloid cells across the three groups before and after abundance matching.

(B) SASP ligand scores in myeloid cells across the three groups in 10 representative analyses randomly selected from 100 abundance-matched repeated analyses.

(C) SASP receptor scores in myeloid cells across the three groups in 10 representative analyses randomly selected from 100 abundance-matched repeated analyses.

(D) Counts of 100 abundance-matched repeated analyses with *p* < 0.05 for CEN vs Control and CO vs Control comparisons of SASP ligand and receptor scores in myeloid cells.

(E) Expression of canonical marker genes across myeloid subsets.

(F) Counts and proportions of myeloid cell subsets.

(G) CCC networks among myeloid subsets: total CCC counts (top), total CCC strength (middle), and combined summary metrics (bottom).

(H) Expression of receptors associated with lost or attenuated signaling in intermediate monocytes.

Asterisks in (B, C and H) denote significance relative to the other two groups: **P* < 0.05, ***P* < 0.01, ****P* < 0.001.

**Figure S4. Divergent intrinsic CCC programs and flow cytometric validation of SASP-related features in myeloid subsets.**

(A, B) Upregulated LR pairs in cDCs, intermediate monocytes, and CD16⁺ monocytes in CEN (A) and CO (B) versus Control, all with *P* < 0.05.

(C) Representative flow cytometry plots showing key target gene expression in myeloid cells from the Control group. Each panel includes: top left, gating strategy; top right, representative CEN sample; bottom left, representative CO sample; bottom right, representative Control sample.

**Figure S5. SASP-related CCC from myeloid cells to EICs in aging population.**(A) t-SNE plot of T cell clusters.

(B) Marker gene expression in T cell subsets.

(C) t-SNE plot of NK cell clusters.

(D) Marker gene expression in NK cell subsets.

(E) LR pairs upregulated in CD8⁺ T cells receiving SASP- or LAG3-related signals from myeloid subsets in CEN *vs*. Control (*P* < 0.05 for all).

(F) LR pairs upregulated in NKT cells receiving SASP- or LAG3-related signals from myeloid subsets in CO *vs*. Control (*P* < 0.05 for all).

(G) Expression of exhaustion markers in EICs, with significance relative to Control: *P* < 0.05 (*), *P* < 0.01 (**), *P* < 0.001 (***)*.*

(H) Flow cytometry sorting plots of major upregulated target genes in EICs from the Control group, arranged as in Figure S4C.

**Figure S6. PRF-related ligand–receptor features and abundance-matched robustness analyses supporting enhanced EIC signaling in healthy aging.**

(A, B) Upregulated LR pairs in CO vs. Control: (A) from NK subsets to CD8⁺ T and NKT cells; (B) from CD8⁺ T and NKT cells to NK subsets.

(C) Numbers of CD8⁺ T cells across the three groups after group-level abundance matching.

(D) Numbers of NK cells across the three groups after group-level abundance matching.

(E) PRF ligand scores in myeloid cells across the three groups in 10 representative analyses randomly selected from 100 abundance-matched repeated analyses.

(F) PRF receptor scores in CD8⁺ T cells across the three groups in 10 representative analyses randomly selected from 100 abundance-matched repeated analyses.

(G) PRF receptor scores in NK cells across the three groups in 10 representative analyses randomly selected from 100 abundance-matched repeated analyses.

(H) Counts of 100 abundance-matched repeated analyses with *p* < 0.05 for CEN vs Control and CO vs Control comparisons of PRF ligand scores in myeloid cells and PRF receptor scores in CD8⁺ T cells and NK cells.

(I) Key regulatory ligands and downstream targets received by NKT cells in healthy aging; red-labeled targets indicate known cytotoxic effectors.

(J) Expression of key ligands, receptors, and target genes in relevant subsets.

Asterisks in (E,F,G and J) denote significance relative to the Control: **P* < 0.05, ***P* < 0.01, ****P* < 0.001.

**Figure S7. Selective PRF-related ligand–receptor features and downstream cytotoxic validation in effector immune cells**

(A) Expression of MHC-I molecules in myeloid cells.

(B, C) Expression of selected receptor genes in NK cells (B) and T cells (C).

(D) Flow cytometry plots of IL-15, IL-18, GZMA, GZMB, and TRAIL in relevant subsets, arranged as in Fig. S2G.

(E) Network diagram of LR pairs upregulated in CO *vs*. Control.

Asterisks in (A–C) denote significance relative to the Control: **P* < 0.05, ***P* < 0.01, ****P* < 0.001.

**Figure S8. Validation in the Hainan dataset.**

(A) t-SNE visualization of PBMC clusters in the Hainan dataset.

(B) Marker gene expression in identified subpopulations.

(C, D) Total CCC counts (C) and overall CCC strength (D) in CEN *vs*. Control (Hainan dataset).

(E) Top 50 LR pairs ranked by interaction strength in the Hainan dataset.

(F) Expression of MHC-I molecules in myeloid cells (*P* < 0.001 for all group comparisons).

(G) Top: exhaustion marker expression in T and NK cells. Bottom: expression of selected receptor genes in T and NK cells (*P* < 0.001 for all group comparisons).

**Table S1. List of Antibodies and Fluorochromes Used for Flow Cytometry.**

| **Antibody** | **Fluorochrome Label** | **Company** | **Catalog number** |
| --- | --- | --- | --- |
| **L/D** | **FVS780** | **BD Pharmingen** | **565388** |
| **CD3** | **BV510** | **BD Pharmingen** | **563109** |
| **CD8** | **BUV395** | **BD Pharmingen** | **563796** |
| **CD14** | **Percp-cy5.5** | **BD Pharmingen** | **562692** |
| **CD16** | **FITC** | **BD Pharmingen** | **555406** |
| **CD56** | **BV786** | **BD Pharmingen** | **564058** |
| **CD1c** | **BV786** | **Biolegend** | **331543** |
| **CLEC10a** | **AF488** | **Thermo** | **MA5-23645** |
| **ICAM1** | **PE-CY7** | **Thermo** | **25-0549-42** |
| **TGF-β1** | **BV421** | **BD Pharmingen** | **562962** |
| **IL-1β** | **BUV395** | **Thermo** | **363-7018-42** |
| **NF-Κb(p65)** | **PE** | **Biolegend** | **653003** |
| **TNF-α** | **BV650** | **BD Pharmingen** | **502938** |
| **CDKN1A(p21)** | **AF647** | **R&D** | **IC1047R-100UG** |
| **IL-15** | **AF700** | **R&D** | **IC2471N-100UG** |
| **IL-18** | **AF700** | **R&D** | **IC2548N-100UG** |
| **GZMA** | **Percp-cy5.5** | **Biolegend** | **507215** |
| **GZMB** | **BV785** | **Biolegend** | **396437** |
| **TRAIL** | **BV650** | **BD Pharmingen** | **743721** |

**Graphical Abstract**

Single-cell profiling of PBMCs from centenarians, their offspring, and elderly controls revealed distinct myeloid-to-effector immune cell communication programs. General aging was characterized by SASP-enriched signaling associated with immunosenescence, whereas healthy aging showed positive immune regulatory signaling associated with enhanced cytotoxicity and immune remodeling.

| ID | Cohort | age | sex | group | WBC（4-10*10^9^/L ） | RBC （4-5.5*10^9/L ） | HB （110-150g/L） | PLT （100-300*10^9/L ） | AST （0-50 U/L） | ALT （0-50U/L） | ALP （30-120U/L） | γ-GT （8-57U/L） | total protein （66-83g/L） | albumin （35-52g/L） | A/G （1.25-2.5） | creatinine （64-104umol/L） | uric acid （208-428umol/L） | cystatin C （0-1.1mg/L） | Total Cholesterol （3.0-5.7mmol/L） | triglyceride （0-1.7mmol/L） | HDLC （1.03-1.55mmol/L） | LDLC （1.89-4.21mmol/L） | AFP （0-8.78ug/L） | CEA （0-5ug/L） | GLU （ 0-6mmol/L） | SBP, mmHg | DBP, mmHg |
| --- | --- | --- | --- | --- | --- | --- | --- | --- | --- | --- | --- | --- | --- | --- | --- | --- | --- | --- | --- | --- | --- | --- | --- | --- | --- | --- | --- |
| CEN-1 | Jiangsu Rugao | 108 | female | CEN | 6.3 | 3.78 | 119 | 140 | 29 | 12 | 114 | 11 | 77.1 | 40.7 | 1.12 | 70 | 194 | 0.96 | 5.2 | 0.92 | 1.82 | 3.11 | 2.84 | 2.5 | 5.4 | 153 | 76 |
| CEN-2 | Jiangsu Rugao | 100 | female | CEN | 3 | 4.03 | 118 | 205 | 20 | 6 | 55 | 11 | 64 | 38.8 | 1.54 | 120 | 372 | 1.5 | 4.8 | 0.73 | 1.61 | 3.02 | 2.74 | 2.7 | 5.6 | 164 | 68 |
| CEN-3 | Jiangsu Rugao | 102 | female | CEN | 5.4 | 4.35 | 127 | 251 | 23 | 12 | 109 | 11 | 70.3 | 37.4 | 1.14 | 118 | 460 | 1.18 | 6.2 | 1.59 | 0.99 | 4.42 | 2.7 | 5.7 | 6.7 | 142 | 72 |
| CEN-4 | Jiangsu Rugao | 103 | male | CEN | 6.6 | 4.15 | 131 | 195 | 14 | 9 | 108 | 23 | 70 | 37.6 | 1.16 | 124 | 480 | 1.95 | 4.9 | 1.76 | 1.24 | 3.21 | 3.09 | 12.4 | 5.2 | 150 | 72 |
| CEN-5 | Jiangsu Rugao | 100 | female | CEN | 5 | 4.16 | 123 | 184 | 25 | 9 | 120 | 14 | 79.7 | 42.4 | 1.14 | 80 | 281 | 1.29 | 5.1 | 1.28 | 1.78 | 3.04 | 4.09 | 6.5 | 5.1 | 122 | 54 |
| GEN-1 | Jiangsu Rugao | 82 | female | CO | 10.9 | 4.02 | 121 | 190 | 27 | 16 | 85 | 10 | 82.4 | 42.3 | 1.05 | 50 | 214 | 0.72 | 4 | 0.75 | 1.46 | 2.49 | 1.37 | 3.8 | 6.3 | 135 | 68 |
| GEN-2 | Jiangsu Rugao | 62 | male | CO | 4.8 | 4.82 | 148 | 154 | 26 | 12 | 92 | 16 | 78.1 | 46.7 | 1.49 | 81 | 304 | 0.86 | 4.6 | 1.41 | 1.51 | 2.66 | 1.45 | 4.3 | 6.8 | 125 | 90 |
| GEN-3 | Jiangsu Rugao | 66 | male | CO | 3.9 | 4.83 | 144 | 96 | 38 | 31 | 111 | 12 | 76.9 | 44.2 | 1.35 | 56 | 323 | 0.73 | 4.1 | 0.76 | 1.28 | 2.47 | 4.95 | 3.5 | 6.7 | 121 | 78 |
| GEN-4 | Jiangsu Rugao | 83 | male | CO | 7.8 | 4.89 | 148 | 290 | 21 | 22 | 114 | 59 | 85.8 | 47 | 1.21 | 95 | 413 | 1.1 | 5.8 | 3.36 | 1.08 | 3.96 | 3.02 | 1.7 | 6.3 | 141 | 76 |
| GEN-5 | Jiangsu Rugao | 61 | male | CO | 4.6 | 4.69 | 161 | 224 | 25 | 12 | 69 | 32 | 75.8 | 45 | 1.46 | 86 | 459 | 0.73 | 5 | 1.24 | 1.44 | 3.27 | 6.03 | 2 | 6 | 144 | 77 |
| NEI-1 | Jiangsu Rugao | 63 | male | control | 5.6 | 4.92 | 154 | 227 | 21 | 14 | 55 | 61 | 77.3 | 45.6 | 1.44 | 65 | 367 | 0.73 | 4.9 | 1.05 | 1.08 | 3.4 | 2.91 | 1.7 | 6.1 | 130 | 81 |
| NEI-2 | Jiangsu Rugao | 65 | female | control | 5 | 4.68 | 140 | 193 | 20 | 19 | 73 | 23 | 81.8 | 41.4 | 1.22 | 58 | 376 | 0.67 | 5.6 | 1.95 | 1.44 | 3.67 | 3.39 | 1.2 | 5 | 142 | 74 |
| NEI-3 | Jiangsu Rugao | 69 | female | control | 4.2 | 4.73 | 137 | 182 | 17 | 11 | 100 | 13 | 75.1 | 42.1 | 1.28 | 61 | 362 | 0.69 | 4.8 | 1.12 | 1 | 3.34 | 5.51 | 1.8 | 5.3 | 150 | 81 |
| NEI-4 | Jiangsu Rugao | 70 | male | control | 6 | 4.53 | 151 | 155 | 52 | 36 | 107 | 31 | 73.4 | 44.9 | 1.58 | 62 | 501 | 0.71 | 3.5 | 2.41 | 1.14 | 2.03 | 3.56 | 2.3 | 5.8 | 140 | 80 |
| NEI-5 | Jiangsu Rugao | 69 | female | control | 4.9 | 4.31 | 133 | 222 | 24 | 19 | 93 | 45 | 75.6 | 43.2 | 1.33 | 70 | 481 | 0.76 | 5.4 | 1.57 | 1.03 | 3.89 | 3.82 | 2.6 | 5.2 | 138 | 85 |
| LG011 | Hainan Sanya | 112 | female | CEN-HN | 5.75 | 4.73 | 122 | 276 | 13 | 4 | 117 | 11 | 71 | 37.5 | 1.1 | 62 | 254 | 1.02 | 3.7 | 0.72 | 1.52 | 1.71 | 1.04 | 1.9 | 6.4 | 126 | 61 |
| LG031 | Hainan Sanya | 112 | female | CEN-HN | 3.04 | 3.68 | 119 | 204 | 37 | 10 | 95 | 6 | 67.5 | 43.1 | 1.8 | 72 | 230 | 1.06 | 5.1 | 1.08 | 1.36 | 3.07 | 2.07 | 6.3 | 6.5 | 130 | 76 |
| LG051 | Hainan Sanya | 107 | male | CEN-HN | 5.51 | 3.81 | 128 | 133 | 30 | 17 | 65 | 25 | 86.1 | 47.8 | 1.2 | 62 | 316 | 0.95 | 5 | 0.97 | 1.58 | 2.77 | 1.83 | 6.4 | 5.7 | 149 | 75 |
| LG013 | Hainan Sanya | 74 | female | control-HN | 7.72 | 4.08 | 123 | 334 | 33 | 23 | 110 | 10 | 82.8 | 45 | 1.2 | 99 | 172 | 1.2 | 6.2 | 1.84 | 1.24 | 4.06 | 2.53 | 3.2 | 5.0 | 125 | 78 |
| LG023 | Hainan Sanya | 74 | female | control-HN | 9.57 | 4.47 | 125 | 314 | 13 | 11 | 71 | 11 | 75.2 | 43.2 | 1.4 | 62 | 219 | 0.66 | 3.9 | 1.19 | 0.93 | 2.31 | 1.33 | 0.9 | 4.5 | 120 | 87 |
| LG032 | Hainan Sanya | 73 | male | control-HN | 6.53 | 4.88 | 149 | 255 | 25 | 27 | 56 | 27 | 77 | 52.1 | 2.1 | 107 | 470 | 1.29 | 6 | 1.7 | 0.91 | 4.07 | 1.88 | 6.7 | 5.7 | 114 | 79 |
